# Comparing the usability of the World Health Organization’s conventional tuberculosis guidelines to the eTB recommendations map: A two-arm superiority randomised controlled trial

**DOI:** 10.1371/journal.pgph.0001166

**Published:** 2022-10-14

**Authors:** Micayla Matthews, Tamara Lotfi, Nancy Santesso, Mark Loeb, Dominik Mertz, Zain Chagla, Anisa Hajizadeh, Thomas Piggott, Bart Dietl, Holger J. Schünemann

**Affiliations:** 1 McMaster University Department of Health Research Methods, Evidence and Impact, Hamilton, Ontario, Canada; 2 McMaster University Michael G. DeGroote Cochrane Canada and GRADE Centre, Hamilton, Ontario, Canada; 3 Department of Medicine, McMaster University, Hamilton, Ontario, Canada; 4 Department of Primary Care, Oxford University, Oxford, United Kingdom; 5 Evidence Prime Incorporated, Hamilton, Ontario, Canada; 6 Department of Biomedical Sciences, Humanitas University, Milano, Italy; PLOS: Public Library of Science, UNITED STATES

## Abstract

Best practices for the dissemination of global health guidelines has not undergone rigorous research. We used a new approach to digitizing World Health Organization (WHO) global tuberculosis guideline recommendations (eTB RecMap) and compared its usability to the conventional method of accessing TB recommendations using the WHO website. We conducted a two-arm superiority randomised controlled trial using a survey among global stakeholders who were past or planned future users of TB guidelines, recommendations, or policy advice. We assigned participants randomly (1:1) to complete an activity using the WHO eTB RecMap or the conventional website. The primary outcome was the accessibility of information and secondary outcomes understanding, satisfaction, and preference for one of the two formats. Between February 26 and August 29, 2021, we received 478 responses from stakeholders, of whom 244 (122 per group) were eligible and provided analysable results. Participants rated the eTB RecMap as more accessible, on average, when compared to the conventional website (on a seven-point scale, the mean difference {MD} was 0.9; 95% confidence interval {CI}: 0.6, 1.2; p < 0.001) and were more likely to correctly answer understanding questions. This is the first trial comparing digitized dissemination formats of health guideline recommendations. Stakeholders rated the WHO eTB RecMap as more accessible than the conventional WHO website for the tested recommendations. They also understood presented information better. The findings support better usability of TB information through the eTB RecMap and contribute to the effort to end the TB epidemic.

**Trial registration:** This trial was registered with ClinicalTrials.gov (NCT04745897) on February 9, 2021.

## Introduction

Tuberculosis (TB) is the leading cause of death from a single infectious agent worldwide, with an estimated 10 million new cases in 2019 [1]. In a concerted effort to end the TB epidemic, the WHO Global TB Programme (WHO-GTB) has issued guidelines with recommendations on TB prevention, diagnosis, treatment, and care [2,3]. Since 2009, these guidelines have been developed using the Grading of Recommendations Assessment, Development and Evaluation (GRADE) method [3], which is a transparent, evidence-based framework for the assessment of the certainty in a body of evidence and recommendation development [4]. Specifically, GRADE assists guideline developers in question prioritization, certainty assessments, balancing benefits and risks, and considering, among other criteria, cost, equity, acceptability, and feasibility in context [4,5].

WHO TB recommendations were located in many discrete publications on the WHO website, including standard, consolidated, interim, and emergency guidelines. Given the potential challenges of navigating through guidelines to find a recommendation, ways to enhance their connectivity and accessibility should be explored like for guidelines in other fields. A mixed-methods study by Hajizadeh, et al. [6] suggested that stakeholders, including members of the WHO-GTB guideline development group (GDG), desire direct access to WHO TB recommendations and supplementary information, such as evidence to decision (EtD) tables and evidence profiles [6].

In an effort to improve usability of WHO TB recommendations, we developed the WHO eTB recommendation map (RecMap) in a collaboration between the WHO Collaborating Center for Infectious Diseases, Research Methods and Recommendations, the Michael G DeGroote Cochrane Canada Centre and the WHO-GTB Programme [6,7]. The RecMap identifies, lists, and maps WHO TB recommendations using recommendation mapping methodology. This methodology builds on the field of evidence mapping by visually organizing recommendations, thus allowing the identification of clusters and gaps [6]. Furthermore, it is anticipated to facilitate the adoption, adaptation, or de novo development of recommendations in a variety of countries and settings through an integrated linkage and access to original content on GRADEpro [8]. The approach has been used, but not tested formally, in other efforts including the eCOVID-19 RecMap to enhance access to all guidelines in general (covid19.recmap.org) and for WHO specifically (who.covid19.recomap.org).

To evaluate if organising recommendations in a recommendation map improves usability, we conducted a randomised controlled trial in users of TB recommendations to evaluate the impact of the new WHO eTB RecMap compared with the conventional method of accessing TB recommendations on accessibility, understanding and satisfaction with the new format.

## Methods

### Study design

This study was a two-arm randomised controlled superiority trial to compare the accessibility of the WHO eTB RecMap (WHO eTB) to the conventional method of accessing TB recommendations through the WHO publications website (WHO TB). A questionnaire was administered using SurveyMonkey (Momentive Inc., California), accessible through a link shared via email. Participants responded to demographic questions and were subsequently randomised using 1:1 allocation to access a recommendation using either WHO eTB or WHO TB (platforms). Participants completed Likert-scale and multiple-choice questions about the platform they were allocated to. After completing the key portion of the trial, they received information about the alternative platform to respond to a question on their preference.

This study was approved by the Hamilton Integrated Research Ethics Board, Hamilton, ON, Canada (7908). No data safety monitoring committee was used. This trial was registered with ClinicalTrials.gov (NCT04745897). The protocol is available in the supporting information (S1 File).

### Participants

Stakeholders who considered themselves to be users or potential users of TB guidelines, recommendations, and policy advice were eligible. We defined a user as someone who responded “yes” to the question “have you ever accessed TB guidelines, recommendations or policy advice in the past?”. A potential user was someone who responded “yes” to the question “do you plan on accessing TB guidelines, recommendations or policy advice in the future?”. Eligible participants could be part of any group with a stake in TB, including the public, healthcare providers, policymakers, and researchers, and there were no restrictions on country of origin, level of education, or prior TB work experience. Individuals who were involved in WHO eTB development were not eligible, and thus were not invited to participate.

We used a targeted snowball recruitment strategy by emailing survey links to WHO TB Guideline Development Group (GDG) members, program managers, and other stakeholders involved in the process of using and applying TB guideline recommendations. We requested that these members disseminate the survey within their networks, which may have included healthcare providers, policymakers, researchers, and people living with TB (S2 File). Furthermore, we shared the survey link with TB, infectious disease, and guideline groups on Twitter and LinkedIn. Participants gave written informed consent upon entry to the survey.

### Randomisation and masking

**Fig 1:**
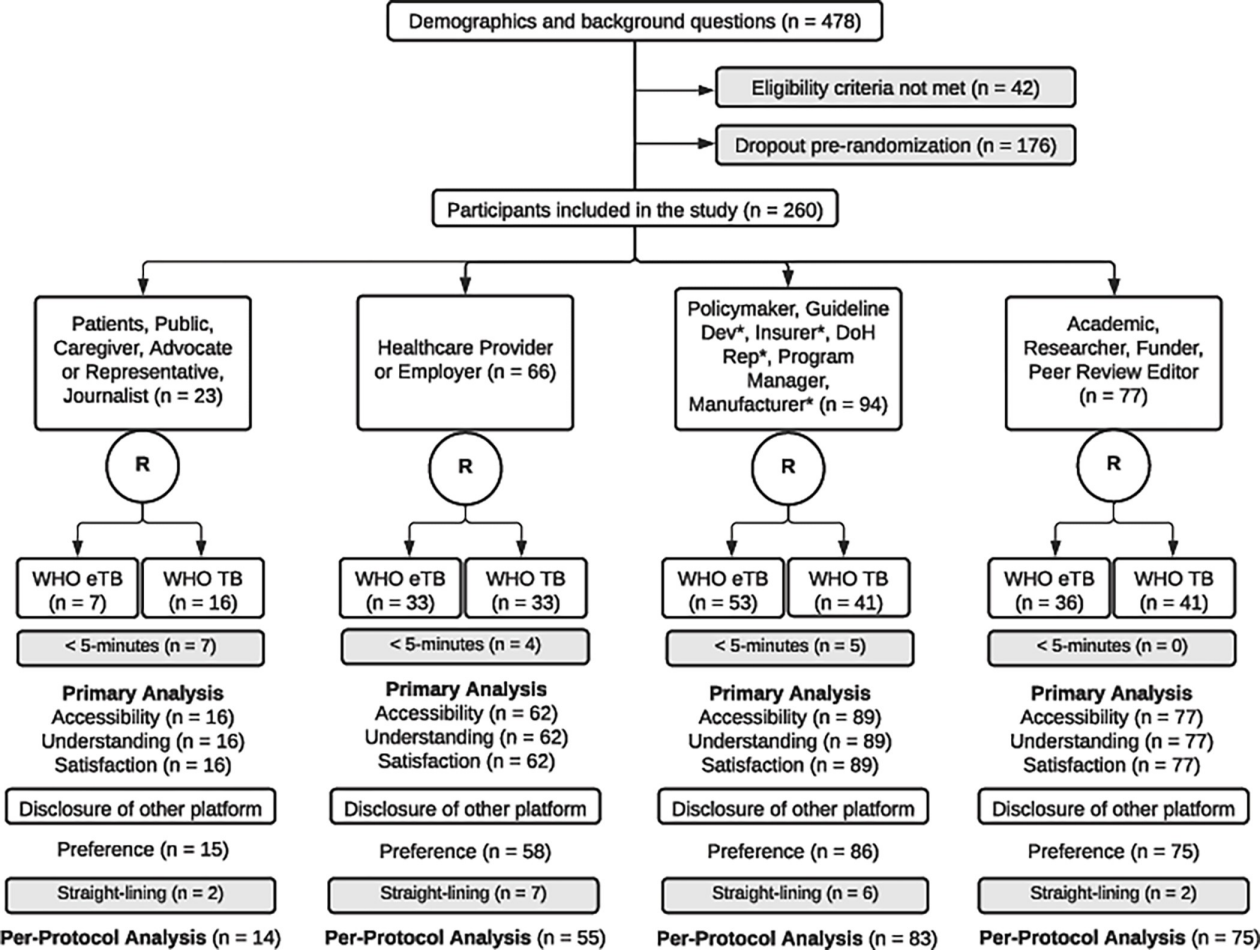
This figure shows the flow of participants through the study. The following abbreviations are included: Guide Dev: Guideline developer; Insurer: Insurer of health services; DoH Rep: Department of health representative; Manufacturer: Drug or device manufacturer.

Participant response to a question on their role as a participant in this study was used for stratification into one of four categories described in Fig 1. Participants within each of these categories were randomly assigned in a 1:1 ratio to the WHO eTB or WHO TB arms. Participants were also randomly assigned to access one of two recommendations in a 1:1 ratio. The same two recommendations were presented for both arms and selected because they contain mostly plain language and were more accessible to non-clinical participants.

The allocation sequence was concealed using SurveyMonkey software based on a commercial, but unknown algorithm without a pre-identified sequence. Participants were not aware of their random allocation to WHO eTB or WHO TB until disclosure (Fig 1). Thus, participants in the control arm were blinded to the eTB RecMap for all outcomes except the secondary outcome of preference. Neutral language was used in both trial arms to prevent promotion of the intervention or comparison.

### Procedures

In this randomised controlled superiority trial, the intervention was the new WHO eTB RecMap (WHO eTB), and the comparison was the conventional method of accessing TB recommendations using the WHO website (WHO TB). We asked survey participants to complete an activity in searching for the same recommendation using the one platform which they had been randomly allocated. Instructions and questions for both arms were worded as similarly as possible. See Table 1 for an overview of the differences between WHO eTB and WHO TB.

**Table 1:** Description of differences between WHO TB and WHO eTB.

	WHO TB	WHO eTB
**Website**	WHO website	WHO eTB website
**Search**	PDF documents	Search bar and filters
**Strength and certainty of evidence defining the recommendation**	Often near recommendation with varying presentation	Always near recommendation presented with consistent language and symbols
**EtD tables presenting evidence underpinning the recommendation**	Found in a separate appendix	Found on the same page with a direct link to the appendix
**Recommendation mapping**	No	Yes

Abbreviations: WHO TB, accessing World Health Organization tuberculosis recommendations via the World Health Organization’s website; eTB, the new eTB recommendations map; PDF, portable document format; EtD, evidence to decision tables.

### Outcomes

This trial used several of the outcomes that have been validated in the evaluation of GRADE Summary of Findings (SoF) tables [9–12]. The primary outcome was the accessibility of information available on the WHO eTB compared to WHO TB. We defined accessibility as the ability to access and use the presented information. This outcome considered the four following domains: (1) how easy it was to find the information (2) how easy it was to understand the information (3) whether the presentation facilitated decision-making (4) whether the website was easy to navigate. We used the original Likert-scale to obtain responses. We used the composite of these four outcomes as primary outcome, and the individual domains were also analyzed as secondary outcomes.

Secondary outcomes were understanding, satisfaction, and preference. We defined understanding as the correct comprehension of findings. This outcome was measured using three multiple-choice questions with five choices and one correct answer. The questions were: ‘what is the recommendation strength?’, ‘what is the certainty of the evidence?’ and ‘on which page does the EtD table for this recommendation start?’. We defined satisfaction as a stakeholder’s impression of platform presentation. This outcome considered the presentation of three domains: (1) home page (2) recommendation list (3) individual recommendation. We used the original Likert-scale to obtain responses. We defined preference as a greater liking of one platform over the other. All participants were provided with a short demonstration of both platforms. They were subsequently asked ‘between the WHO Tuberculosis Guidelines (current website), and the WHO eTB Guidelines (alternative website), which do you prefer?’. We measured preference using a Likert-type scale to express the degree of preference with seven answer options (1 = strongly prefer WHO TB, 2 = prefer WHO TB, 3 = somewhat prefer WHO TB, 4 = same preference for WHO TB and eTB, 5 = somewhat prefer WHO eTB, 6 = prefer WHO eTB, 7 = strongly prefer WHO eTB).

### Statistical analysis

For this two-sided (α = 0.05) superiority analysis, sample size was calculated using the primary outcome of accessibility in WINPEPI (PEPI-for-Windows) version 11.65. With a sample size of 122 per arm (244 total) we would achieve 80% power to detect a difference on the Likert-scale of 0.5 with a standard deviation of 1.0 between intervention and control groups, generally considered an important change on the seven-point Likert scale and observed in previous studies measuring on GRADE SoF tables [9–13]. We assumed that 15% of those starting would not complete the survey, but we did not factor stratifying participants by stakeholder group into the calculation, as the aim of stratification was to balance participants rather than be sufficiently powered to detect subgroup effects.

We summarized participant baseline characteristics and outcomes using means and standard deviations (SD) for continuous variables, and proportions for categorical variables. We performed a primary analysis including all randomised participants, except for those who completed the survey in less than five minutes. We determined this cut-off a priori as part of the protocol because user testing determined that it would be impossible to comprehend and complete the work in that time. We conducted a second per-protocol analysis excluding participants who were flagged by pre-defined but unknown SurveyMonkey software for poor-quality responses known as straight-lining. Straight-lining is defined by SurveyMonkey as responses to questions with the same answer option or pattern. Participants flagged for straight-lining, as well as those who spent less than five minutes on the survey, were removed from the per-protocol analysis.

For the outcomes of accessibility and satisfaction, we used t-tests and mean differences 95% confidence intervals (95% CIs) to compare means and standard deviations (SDs) between the intervention and control groups. For the outcome of understanding, we used χ^2^ tests and risk difference (RD) with 95% CIs to compare the proportion of correct responses between groups. For preference, we presented preference as mean (SD) overall and for both trial arms. Skewness, Shapiro-Wilk tests, and histograms were used to evaluate whether the distribution was shifted toward the same preference in both groups. Levene’s test of equal variances was used for all t-tests and degrees of freedom were adjusted for p < 0.05. We conducted the analyses using IBM SPSS (Statistical Package for Social Sciences) version 23.

We performed a pre-planned interim analysis with data collected between February 26 and March 24, 2021, based on the thesis defence date of the first author (MM). The interim analysis was not intended or used to stop the study or draw final conclusions (S3 File).

## Results

Between February 26 and August 29, 2021, 478 participants enrolled in the study. Of these, 176 dropped out prior to randomisation and 42 did not satisfy the eligibility criteria. A total of 260 participants were randomised. Of these, 16 were removed for less than five-minute completion time, leaving 244 for the primary analysis. In this sample, 48% (117/244) were female, 91% (223/244) were between the ages of 26 and 65, 63% (153/244) worked or lived in a low-and middle-income country (LMIC), 26% (63/244) in a high-income country (HIC), and 11% (26/244) in both. Most participants (79%; 193/244) held a professional or graduate degree, and at least three years (77%; 189/244) of TB-related work experience. Most participants also considered themselves to be comfortable (26%; 64/244) or very comfortable (56%; 136/244) with basic information and communication technologies. Participant strata comprised of 7% (16/244) in the patient group, 25% (62/244) in the healthcare provider group, 36% (89/244) in the policymaker group, and 32% (77/244) in the academic group. We found no differences in study outcomes between strata on stakeholder roles, so Table 2 describes non-stratified data per group.

**Table 2:** Baseline characteristics of participants per group.

	WHO eTB (n = 122)	WHO TB (n = 122)
**Gender: n (%)**		
Female	58 (47)	59 (48)
Male	63 (52)	60 (49)
Other	-	1 (1)
Prefer not to respond	1 (1)	2 (2)
**Age (years): n (%)**		
< 25	3 (3)	7 (6)
26–35	41 (34)	29 (24)
36–45	37 (30)	37 (30)
46–55	25 (20)	29 (24)
56–65	10 (8)	15 (12)
66–75	4 (3)	5 (4)
Prefer not to respond	2 (2)	-
**Setting: n (%)**		
HIC	29 (24)	34 (28)
LMIC	76 (62)	77 (63)
HIC and LMIC	16 (13)	10 (8)
Prefer not to respond	1 (1)	1 (1)
**Education: n (%)**		
Primary	-	1 (1)
High school	-	2 (2)
College	2 (2)	6 (5)
Bachelor	18 (15)	21 (17)
Professional	18 (15)	27 (22)
Graduate	50 (40)	38 (31)
Professional and graduate	33 (27)	27 (22)
Prefer not to respond	1 (1)	-
**TB work (years): n (%)**		
< 1	6 (5)	12 (10)
1–2	14 (12)	8 (7)
3–5	20 (16)	16 (13)
6–9	17 (14)	12 (10)
> 10	59 (48)	65 (53)
Not applicable	4 (3)	5 (4)
Prefer not to respond	2 (2)	4 (3)

Abbreviations: WHO, World Health Organization; TB, Tuberculosis; HIC, high income country; LMIC, low- and middle-income country.

Across four domains, participants assigned to the new WHO eTB RecMap rated the information as more accessible compared to the conventional WHO TB website (MD on the seven-point scale: 0·9; 95% CI: 0·6, 1·2; p < 0.001). The largest mean differences were observed for the statements “it was easy to find the information” (MD 1.1; 95% CI: 0.7, 1.5; p < 0.001) and “this website was easy to navigate” (MD 1.3; 95% CI: 0.9, 1.7; p < 0·001). Participants assigned to WHO eTB also stated that it was easier to understand the information (MD 0.6; 95% CI: 0.3, 0.9; p = 0.001) and that the information was presented in a way that would help them make a decision (MD 0.7; 95% CI: 0.3, 1.0; p < 0.001, Table 3).

**Table 3:** Overall accessibility of information [mean (SD)].

	WHO eTB^a^ (n = 122)	WHO TB^a^ (n = 122)	MD (95% CI)^b^ p value
**Overall Accessibility** ^**c**^	**5.6 (1.0)**	**4.7 (1.5)**	**0.9 (0.6, 1.2)** < 0.001
It was easy to find the information	5.6 (1.1)	4.4 (1.9)	1.1 (0.7, 1.5) < 0.001
This website was easy to navigate	5.6 (1.2)	4.3 (1.8)	1.3 (0.9, 1.7) < 0.001
It was easy to understand the information	5.6 (1.0)	5.0 (1.6)	0.6 (0.3, 0.9) 0.001
The information was presented in a way that would help me make a decision	5.7 (1.0)	5.0 (1.5)	0.7 (0.3, 1.0) < 0.001

Abbreviations: SD, standard deviation; WHO, World Health Organization; TB, tuberculosis; MD, mean difference; CI, confidence interval.

^a^ Likert-scale from 1 = strongly disagree to 7 = strongly agree.

^b^ Equal variances could not be assumed using Levene’s test, degrees of freedom adjusted.

^c^ Composite of four domains (primary outcome).

Participants assigned to the WHO eTB were more likely to correctly answer understanding questions: risk difference of 47% (95% CI 37, 57; p < 0.001, Table 4) for correct responses to the EtD question. There were also 20% differences (95% CI 8, 32) in correct responses to questions on recommendations strength (p = 0.002) and certainty of evidence (p = 0.001).

**Table 4:** Percentage # (%) of participants who responded correctly to understanding questions.

	WHO eTB (n = 122)	WHO TB (n = 122)	Risk Difference (95% CI) p value^a^
On which page does the evidence to decision (EtD) table for this recommendation start?	66/122 (54)	9/122 (7)	47 (37, 57) < 0.001
What is the recommendation strength?	88/122 (72)	64/122 (52)	20 (8, 32) 0.002
What is the certainty of evidence?	66/122 (54)	41/122 (34)	20 (8, 32) 0.001

^a^ Pearson’s chi-square.

Participants assigned to WHO eTB were more satisfied with the presentation of the home page (MD 1.0; 95% CI: 0.7, 1.4; p < 0.001, Table 5) and individual recommendations page (MD 0.3; 95% CI: 0.002, 0.6; p = 0.048) compared to WHO TB. We observed that participants assigned to WHO eTB also appeared more satisfied with the list of recommendations page, but the difference was smaller than for the other outcomes (MD 0.2; 95% CI: -0.1, 0.5; p = 0.214).

**Table 5:** Satisfaction with the presentation of platform pages [mean (SD)].

	WHO eTB^a^ (n = 122)	WHO TB^a^ (n = 122)	MD (95% CI) p value
Home page	5.6 (1.1)	4.6 (1.8)	1.0 (0.7, 1.4)^b^ < 0.001
Individual recommendation	5.6 (1.1)	5.3 (1.3)	0.3 (0.002, 0.6) 0.048
List of recommendations	5.5 (1.1)	5.3 (1.3)	0.2 (-0.1, 0.5)^b^ 0.214

Abbreviations: SD, standard deviation; WHO, World Health Organization; TB, tuberculosis; MD, mean difference; SE, standard error.

^a^ Likert-scale from 1 = very dissatisfied to 7 = very satisfied.

^b^ Equal variances could not be assumed using Levene’s test, degrees of freedom adjusted.

Overall, participants (n = 234) “somewhat preferred WHO eTB” (4.8; SD 1.8), after reviewing demonstrations of both platforms. This preference was stronger in participants who were assigned to first review the WHO eTB (5.0; SD 1.6) compared to those who first reviewed the WHO TB (4.5; SD 2.0) (p = 0.044) platform. Both arms were left-skewed toward this preference (p < 0.001).

There were no differences between the primary and per-protocol analyses that excluded participants suspected of straight lining (responses to questions with the same answer option or pattern) (n = 227) (S3 File).

## Discussion

The primary aim of this RCT was to determine if the WHO eTB RecMap [6,7] improved the usability of WHO TB recommendations for stakeholders of interest. Participants represented a diverse group of users and potential users of TB recommendations. Our results suggest that the WHO eTB RecMap improves the usability of these recommendations for stakeholders when compared to the conventional method. Specifically, participants found, on average, that the information presented in WHO eTB was easier to find, easier to understand, that it was presented in a way that would help them make a decision, and that the website was easier to navigate. We sought to corroborate accessibility with the secondary outcome of understanding, and we found that the eTB RecMap improved the ability of participants to identify recommendation strength and certainty, and to access the supporting evidence and decisions underpinning the recommendation (EtD). Furthermore, stakeholders were more satisfied with the presentation of the WHO eTB home page and presentation of individual recommendations, although the effects were small. Overall, participants somewhat preferred the eTB RecMap to the conventional WHO TB website.

To our knowledge, this is the first RCT comparing stakeholder feedback on the presentation of two guideline platforms. However, several studies have explored stakeholder perceptions of guideline development and presentation, as well as the factors that influence their uptake. One qualitative study by Fearns, et al. [14] explored public perceptions of clinical practice guidelines and found that participants desired information to help them make decisions, but current numerical formats may not always be accessible to a public audience. Additionally, a content analysis by Santesso, et al. [15] found that patient versions of guidelines may not always address stakeholder needs, as they rarely include important EtD information, such as beliefs, values and preferences, accessibility, costs and feasibility. Furthermore, a realist review by Kastiner, et al. [16] which sought to identify the factors associated with guideline uptake, found that effective communication of content, including simple, clear and persuasive language, improved the implementability of guidelines by stakeholders.

This trial expands on the methods of previous RCTs used to evaluate the presentation of guideline information, specifically, comparing new GRADE SoF tables to conventional formats. These studies also evaluated the outcomes of participant understanding, accessibility, satisfaction and preference [9,11,12]. Akl, et al. [17] found that participants demonstrated a better understanding of strength of recommendations and quality of evidence when this information was presented as symbols, rather than numbers. Carrasco-Labra, et al. [9] and Vandvik, et al. [12] identified that stakeholders preferred the presentation of risk differences over absolute risk estimates, as well as the inclusion of narrative statements to supplement numerical data. Similar, to our trial, these studies objectively evaluated perceptions of new to conventional formats to identify areas of improvement.

This study had several strengths. First, we used a randomised design reported in accordance with the CONSORT statement on randomised trials, which reduces the risk of confounding, selection and reporting bias (S1 Table) [18,19]. Second, we used several previously validated outcomes from similar trials [9–12]. Third, we gathered feedback from diverse group of stakeholders, thus improving the generalizability of findings using a carefully developed approach to presenting recommendations [6]. Nevertheless, the snowball recruitment strategy through established guideline networks may limit the generalizability in potential users. This study has some additional limitations. First, the ability to blind participants was limited, as some may have been aware of eTB development. We consider this probability to be small, as the main publication and awareness campaigns began after the majority of participants had been recruited. Our pre-planned a priori interim analysis that coincided with publication of the eTB platform did not suggest differences in the results before and after these campaigns (S3 File). Second, data were collected using an online survey and, thus, there was limited control over the environment in which the survey was performed. Third, participants often claimed to be part of more than one stakeholder group (e.g., a healthcare provider involved in research), but they were required to select just one for stratification. Fourth, we did not power our trial to conclusively evaluate results by participant strata. Fifth, some of the secondary outcomes showed mean effects that were below the minimal important difference (MID) although the CI of the effects crossed the MID. Despite these small effects, a proportion of users would still experience benefit from the eTB platform presentation related to these secondary outcomes [20]. Furthermore, to improve the presentation on the eTB platform, we have made additional changes to the final presentation on the eTB RecMap and our new eCOVID RecMap that catalogues recommendations related to COVID-19 guidelines [21].

Tuberculosis guideline recommendations developed by the WHO assist stakeholders in making evidence-informed decisions on TB prevention, diagnosis, treatment, and care [2,3]. According to the Guidelines International Network (G-I-N) and the National Institute for Health and Care Excellence (NICE), stakeholder engagement is important to ensuring that guideline products are feasible and acceptable to end-users [22,23]. This study engaged stakeholders in evaluating the presentation of recommendations through the outcomes of accessibility, understanding, and satisfaction, which are surrogates for the correct implementation of evidence in practice. Thus, our findings suggest that the new WHO eTB RecMap will help stakeholders make evidence-informed decisions on TB in support of the WHO End TB strategy [2]. This study demonstrates that RCTs may be used to compare stakeholder feedback on guideline platforms. Future studies should seek to explain the findings of this trial through qualitative and user testing techniques, such as the study by Rosenbaum, et al. for SoF tables [10]. Furthermore, additional trials should focus on specific stakeholder groups, such as patients and the public, to determine optimal ways to present recommendations. There are three trials underway that test the use of plain language recommendations presented as part of a recommendation map for COVID-19 (https://clinicaltrials.gov/ct2/show/NCT05358990).

In conclusion, the new WHO eTB RecMap [6,7] improved the usability of WHO TB recommendations and supporting evidence for stakeholders of interest. Our findings support the continued use, promotion, and quality improvement of WHO eTB. Researchers should consider the use of RCTs to evaluate stakeholder feedback on guideline presentation in the future. The RecMap approach has been used for COVID-19 (covid19.recmap.org and who.covid19.recmap.org) and is being implemented by the PanAmerican Health Organization across all guidelines that use the GRADE approach (https://bigg-rec.bvsalud.org/en). This trial provides some evidence that this may be a wise decision.

## Supporting information

S1 Table CONSORT checklist. (PDF)

S1 File Trial protocol. (PDF)

S2 File Consent, survey, and recruitment details. (PDF)

S3 File Interim and per-protocol analysis details. (PDF)
