# Supplementary material for: Comparing the usability of the World Health Organization’s conventional tuberculosis guidelines to the eTB recommendations map: A two-arm superiority randomised controlled trial
Source: PLOS Glob Public Health. 2022 Oct 14;2(10):e0001166. doi: 10.1371/journal.pgph.0001166 (PMC10021182; doi:10.1371/journal.pgph.0001166)
Supplement: S1 Table — (PDF) [file pgph.0001166.s001.pdf]

S1 Table. CONSORT checklist.

CONSORT 2010 checklist of information to include when reporting a randomised trial [1]

Section/Topic	Item No	Checklist item	Reported on page No
**Title and abstract**			
	1a	Identification as a randomised trial in the title	Page 1
	1b	Structured summary of trial design, methods, results, and conclusions	Page 2
**Introduction**			
Background and objectives	2a	Scientific background and explanation of rationale	Page 3
	2b	Specific objectives or hypotheses	Page 4
**Methods**			
Trial design	3a	Description of trial design (such as parallel, factorial) including allocation ratio	Page 4
	3b	Important changes to methods after trial commencement (such as eligibility criteria), with reasons	N/A
Participants	4a	Eligibility criteria for participants	Page 5
	4b	Settings and locations where the data were collected	Page 5
Interventions	5	The interventions for each group with sufficient details to allow replication, including how and when they were actually administered	Page 6
Outcomes	6a	Completely defined pre-specified primary and secondary outcome measures, including how and when they were assessed	Page 7
	6b	Any changes to trial outcomes after the trial commenced, with reasons	N/A
Sample size	7a	How sample size was determined	Page 8
	7b	When applicable, explanation of any interim analyses and stopping guidelines	Page 9
Randomisation:			
Sequence generation	8a	Method used to generate the random allocation sequence	Page 6
	8b	Type of randomisation; details of any restriction (such as blocking and block size)	Page 6
Allocation concealment mechanism	9	Mechanism used to implement the random allocation sequence (such as sequentially numbered containers), describing any steps taken to conceal the sequence until interventions were assigned	Page 6
Implementation	10	Who generated the random allocation sequence, who enrolled participants, and who assigned participants to interventions	Page 6
Blinding	11a	If done, who was blinded after assignment to interventions (for example, participants, care providers, those assessing outcomes) and how	Page 6
	11b	If relevant, description of the similarity of interventions	Page 6
Statistical methods	12a	Statistical methods used to compare groups for primary and secondary outcomes	Page 8
	12b	Methods for additional analyses, such as subgroup analyses and adjusted analyses	Page 8
**Results**			

S1 Table. CONSORT checklist.

Participant flow (a diagram is strongly recommended)	13a	For each group, the numbers of participants who were randomly assigned, received intended treatment, and were analysed for the primary outcome	Page 10
	13b	For each group, losses and exclusions after randomisation, together with reasons	Page 10
Recruitment	14a	Dates defining the periods of recruitment and follow-up	Page 10
	14b	Why the trial ended or was stopped	N/A
Baseline data	15	A table showing baseline demographic and clinical characteristics for each group	Page 11
Numbers analysed	16	For each group, number of participants (denominator) included in each analysis and whether the analysis was by original assigned groups	Page 10
Outcomes and estimation	17a	For each primary and secondary outcome, results for each group, and the estimated effect size and its precision (such as 95% confidence interval)	Page 11
	17b	For binary outcomes, presentation of both absolute and relative effect sizes is recommended	Page 10
Ancillary analyses	18	Results of any other analyses performed, including subgroup analyses and adjusted analyses, distinguishing pre-specified from exploratory	Page 10
Harms	19	All important harms or unintended effects in each group (for specific guidance see CONSORT for harms)	N/A
**Discussion**			
Limitations	20	Trial limitations, addressing sources of potential bias, imprecision, and, if relevant, multiplicity of analyses	Page 14
Generalisability	21	Generalisability (external validity, applicability) of the trial findings	Page 14
Interpretation	22	Interpretation consistent with results, balancing benefits and harms, and considering other relevant evidence	Page 15
**Other information**			
Registration	23	Registration number and name of trial registry	Page 2,5
Protocol	24	Where the full trial protocol can be accessed, if available	Page 18
Funding	25	Sources of funding and other support (such as supply of drugs), role of funders	Page 17

Items to include when reporting a randomized trial in a journal or conference abstract [2]

**Item**	**Description**	**Reported on line number**
Title	Identification of the study as randomized	Page 1
Authors *	Contact details for the corresponding author	N/A
Trial design	Description of the trial design (e.g. parallel, cluster, non-inferiority)	Line 26
Methods		
Participants	Eligibility criteria for participants and the settings where the data were collected	Line 27
Interventions	Interventions intended for each group	Line 29
Objective	Specific objective or hypothesis	Line 30
Outcome	Clearly defined primary outcome for this report	Line 30
Randomization	How participants were allocated to interventions	Line 28
Blinding (masking)	Whether or not participants, care givers, and those assessing the outcomes were blinded to group assignment	Line 28

S1 Table. CONSORT checklist.

Results		
Numbers randomized	Number of participants randomized to each group	Line 32
Recruitment	Trial status	Line 41
Numbers analysed	Number of participants analysed in each group	Line 32
Outcome	For the primary outcome, a result for each group and the estimated effect size and its precision	Line 33
Harms	Important adverse events or side effects	N/A
Conclusions	General interpretation of the results	Line 37
Trial registration	Registration number and name of trial register	Line 41
Funding	Source of funding	N/A

**this item is specific to conference abstracts*

References

1. Moher D, Hopewell S, Schulz KF, et al. CONSORT 2010 Explanation and Elaboration: updated guidelines for reporting parallel group randomised trials. *BMJ*. 2010;**340**. doi:10.1136/bmj.c869

2. Hopewell S, Clarke M, Moher D, et al. CONSORT for reporting randomised trials in journal and conference abstracts. *Lancet*, 2008,**371**(9609);281-3. doi:10.1016/S0140-6736(07)61835-2
