# Supplementary material for: Comparing the usability of the World Health Organization’s conventional tuberculosis guidelines to the eTB recommendations map: A two-arm superiority randomised controlled trial
Source: PLOS Glob Public Health. 2022 Oct 14;2(10):e0001166. doi: 10.1371/journal.pgph.0001166 (PMC10021182; doi:10.1371/journal.pgph.0001166)
Supplement: S1 File — (PDF) [file pgph.0001166.s002.pdf]

Comparing the Accessibility of the World Health Organization's Conventional Tuberculosis Guidelines to the eTB Recommendations Map: A Two-Arm Superiority Randomised Controlled Trial (Protocol)

Authors:
Micayla Matthews[1,2], Tamara Lotfi[1,2], Nancy Santesso[1,2], Mark Loeb[1,2], Dominik Mertz[1,3], Zain Chagla[1,3], Anisa Hajizadeh[1,4], Thomas Piggott[1], Bart Dietl[5], Holger J. Schünemann[1,2]

Institutional Affiliations:
(1) McMaster University Department of Health Research Methods, Evidence and Impact, Hamilton ON, Canada (2) McMaster University Michael G. DeGroote Cochrane Canada and GRADE Centre, Hamilton ON, Canada (3) Department of Medicine, McMaster University, Hamilton ON, Canada (4) Department of Primary Care, Oxford University, Oxford, United Kingdom (5) Evidence Prime Incorporated, Hamilton, Ontario, Canada

Addresses:
(1 & 2) McMaster University Medical Centre, 1280 Main Street West, 2C Area, Hamilton, Ontario, L8S 4K1, Canada (3) Department of Medicine McMaster University Health Sciences Centre, 4V33 1200 Main Street West Hamilton, Ontario, L8N 3Z5, Canada (4) Radcliffe Primary Care Building, Radcliffe Observatory Quarter, Woodstock Rd, Oxford, OX2 6GG, United Kingdom (5) Evidence Prime Inc., 175 Longwood Rd S, Hamilton, Ontario, L8P 0A1, Canada

Corresponding Author:
Holger Schünemann, Michael G DeGroote Cochrane Canada and McMaster GRADE centres; Department of Health Research Methods, Evidence and Impact, McMaster University, HSC-2C, 1280 Main St West; Hamilton, ON L8N 3Z5, Canada; Tel: +1 9055259140, E-mail: schuneh@mcmaster.ca

Abstract

Objective: In an effort to make tuberculosis (TB) guidelines issued by the World Health Organization (WHO) more accessible for stakeholders, we developed a new electronic map of tuberculosis recommendations (eTB RecMap). The objective of this study is to compare the accessibility of the new eTB RecMap to the conventional method of accessing TB recommendations using the WHO website.

Study Design: We will conduct a two-arm superiority randomised controlled trial among stakeholders who were past or planned future users of TB guidelines. Using a 1:1 ratio, we will randomly assign participants to complete an activity using the WHO eTB RecMap or the conventional website. We will compare the outcomes of accessibility, understanding, satisfaction and preference between groups.

Keywords
Guideline; Recommendation; Tuberculosis; GRADE; Evidence-Based Practice; Evidence to Decision Table

Trial Registration
This trial is registered with ClinicalTrials.gov (NCT04745897).

Funding
The development of the eTB RecMap was funded by the WHO. This trial was not funded by the WHO, and the WHO will not be involved in data collection or analysis of study findings.

Consent to Participate
The study questionnaire includes a consent statement that described the study purpose, confidentiality, risks, and voluntary participation.

Data Protection and Confidentiality
Anonymized survey data will be stored in SurveyMonkey® password-protected software. Participant names collected for follow-up will be stored in a separate document on a secure password-protected computer. Only the research team will have access to these data.

Ethics Approval
Hamilton Integrated Research Ethics Board (HiREB)

Abbreviations

EtD: Evidence to Decision
GDG: Guideline Development Group
G-I-N: Guidelines International Network
GRADE: Grading of Recommendations Assessment, Development and Evaluation
HIC: high-income country
LMIC: low- and middle-income country
NICE: National Institute for Health and Care Excellence
RCT: Randomised Controlled Trial
eTB RecMap: Electronic Map of Tuberculosis Recommendations
SoF: Summary of Findings
TB: Tuberculosis
UN: United Nations
WHO: World Health Organization
WHO GTB: WHO Global TB Programme

Background

Tuberculosis (TB) is the leading cause of death from a single infectious agent worldwide, with an estimated 10 million new cases in 2019.[1] In a concerted effort to end the TB epidemic, the WHO Global TB Programme (WHO-GTB) has issued guidelines with recommendations on TB prevention, diagnosis, treatment, and care.[2,3] Since 2009, these guidelines have been developed using the Grading of Recommendations Assessment, Development and Evaluation (GRADE) method[3], which is a transparent, evidence-based framework for the assessment of the certainty in a body of evidence and recommendation development.[4] Specifically, GRADE assists guideline developers in question prioritization, certainty assessments, balancing benefits and risks, and considering, among other criteria, cost, equity, acceptability, and feasibility in context.[4,5]

WHO TB recommendations are located in many discrete publications on the WHO website, including standard, consolidated, interim, and emergency guidelines. Given the large number and variety of publications, ways to enhance their connectivity and accessibility should be explored like for guidelines in other fields. A mixed-methods study by Hajizadeh, et al.[6] suggested that stakeholders, including members of the WHO-GTB guideline development group (GDG), desire direct access to WHO TB recommendations and supplementary information, such as evidence to decision (EtD) tables and evidence profiles.[6]

In an effort to improve accessibility and use of WHO TB recommendations, we developed a WHO eTB recommendation map (RecMap) in a collaboration between the WHO Collaborating Center for Infectious Diseases, Research Methods and Recommendations, the Michael G DeGroote Cochrane Canada Centre and the WHO-GTB.[6,7] This RecMap identifies, lists, and maps WHO TB recommendations using recommendation mapping methodology. This methodology visually organizes recommendations, thus allowing the identification of clusters and gaps.[6] Furthermore, it is anticipated to facilitate the adoption, adaptation, or de novo development of recommendations in a variety of countries and settings through an integrated linkage and access to original content on GRADEpro.[8]

In order to compare the accessibility of the new WHO eTB RecMap with the conventional method of accessing TB recommendations using the WHO website, we will conduct a randomised controlled trial.

Methods

This study will be reported in accordance with the most recent guidance from the Consolidated Standards of Reporting Trials (CONSORT).[9] We registered the trial with ClinicalTrials.gov (trial registration: NCT04745897).

Study Setting

This study was a two-arm randomised controlled superiority trial to compare the accessibility of the WHO eTB RecMap (WHO eTB) to the conventional method of accessing TB recommendations through the WHO publications website (WHO TB). It will be administered

using a SurveyMonkey® questionnaire, accessible through a link shared via email. Participants will respond to demographic questions and will subsequently be randomised using 1:1 allocation to access a recommendation using either WHO eTB or WHO TB (platforms). Randomization is stratified by participant background (e.g. patient, healthcare provider, policymaker) in order to ensure balance between groups. Participants will complete Likert-scale and multiple-choice questions about the platform which they were allocated. After completing the key portion of the trial, they will receive information about the alternative platform to respond to a question on their preference.

Figure 1. Survey RCT Flow Diagram

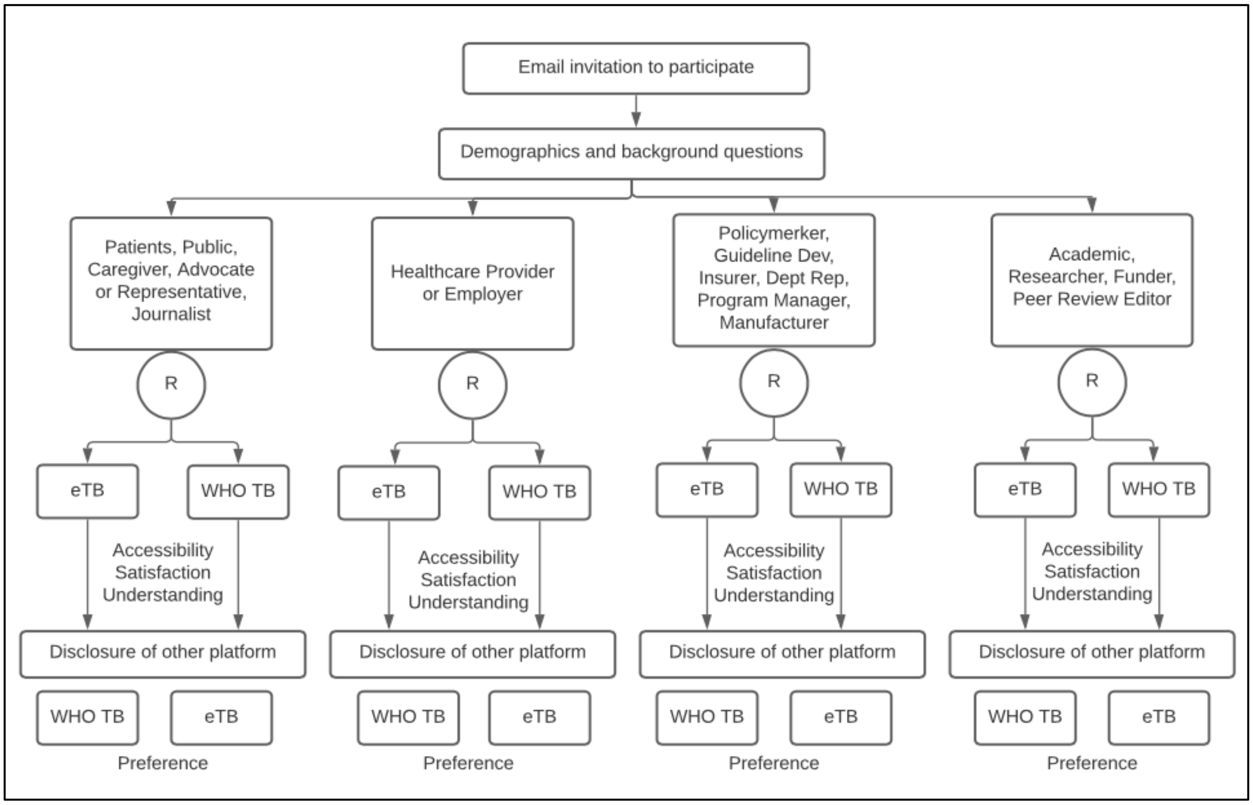

Participants

Eligibility Criteria

Stakeholders who consider themselves to be users or potential users of TB guidelines, recommendations, and policy advice are eligible. For the purposes of this trial, we define a user as someone who responds "yes" to the question "have you ever accessed TB guidelines, recommendations or policy advice in the past?". A potential user is someone who responds "yes" to the question "do you plan on accessing TB guidelines, recommendations or policy advice in the future?". Eligible participants could be part of any group with a stake in TB, including the public, healthcare providers, policymakers, and researchers, and there are no restrictions on

country of origin, level of education, or prior TB work experience. Individuals who were involved in WHO eTB development are not eligible, thus will not be invited to participate.

Recruitment

We will use a targeted snowball recruitment strategy by emailing survey links to WHO TB Guideline Development Group (GDG) members, program managers, and other stakeholders involved in the process of using and applying TB guideline recommendations. We will request that these members disseminate the survey within their networks, which may have included healthcare providers, policymakers, researchers, and people living with TB. Furthermore, we plan to share the survey link with TB, infectious disease, and guideline groups on the social media platforms Twitter and LinkedIn.

Intervention and Comparison

In this randomised controlled superiority trial, the intervention is the new WHO eTB RecMap (WHO eTB), and the comparison is the conventional method of accessing TB recommendations using the WHO website (WHO TB). We will ask survey participants to complete an activity in searching for the same recommendation using the one platform which they had been randomly allocated. Instructions and questions for both arms are worded as similarly as possible. See Table 1 for an overview of the differences between WHO eTB and WHO TB.

Table 1. Description of differences between WHO TB and WHO eTB

	**WHO TB**	**WHO eTB**
Website	WHO website	WHO eTB website
Search	PDF documents	Search bar and filters
Strength and certainty defining the recommendation	Often near recommendation	Always near recommendation
EtD tables	Separate appendix	Link to page in appendix
Recommendation mapping	No	Yes

Abbreviations: WHO TB, accessing World Health Organization tuberculosis recommendations via the World Health Organization's website; eTB, the new eTB recommendations map; PDF, portable document format; EtD, evidence to decision tables

Outcomes

This trial uses several of the outcomes that have been validated in the evaluation of GRADE Summary of Findings (SoF) tables and are defined as follows.[10–13]

Primary Outcome

Accessibility of Information

The primary outcome of interest is the accessibility of information available on the WHO eTB compared to WHO TB. We define accessibility as the ability to access and use the presented information. This outcome considers the four following domains: (1) how easy it was to find the

information (2) how easy it was to understand the information (3) whether the presentation facilitated decision-making (4) whether the website was easy to navigate. We will use the composite of these four outcomes as primary outcome (see statistical analysis), and the individual domains will also be analyzed as secondary outcomes.

Secondary Outcomes

Understanding

We define understanding as the correct comprehension of findings. This outcome will be measured using three multiple-choice questions with five choices and one correct answer. The questions are: 'what is the recommendation strength?', 'what is the certainty of the evidence?' and 'on which page does the EtD table for this recommendation start?'.

Satisfaction

We define satisfaction as a stakeholder's impression of platform presentation. This outcome considers the presentation of three domains: (1) home page (2) recommendation list (3) individual recommendation.

Preference

We define preference as a greater liking of one platform over the other. All participants will be provided with a short demonstration of both platforms. They will subsequently be asked 'between the WHO Tuberculosis Guidelines (current website), and the WHO eTB Guidelines (alternative website), which do you prefer?'.

Outcomes Measurement

We will use the original Likert-scale to obtain responses for the outcomes of accessibility and satisfaction. We will measure preference using a Likert-type scale to express the degree of preference with seven answer options (1 = strongly prefer WHO TB, 2 = prefer WHO TB, 3 = somewhat prefer WHO TB, 4 = same preference for WHO TB and eTB, 5 = somewhat prefer WHO eTB, 6 = prefer WHO eTB, 7 = strongly prefer WHO eTB).

Sample Size Calculation

Sample size was calculated using the primary outcome of accessibility in WINPEPI® (PEPI-for-Windows) version 11.65. For this two-sided ($\alpha = 0.05$) superiority analysis, these computations were made based on a t-test with the null hypothesis that there is no difference between the WHO eTB and WHO TB in the accessibility of information.

H_0: *WHO eTB* = *WHO TB*
H_1: *WHO eTB* ≠ *WHO TB*

With sample sizes of 122 per arm (244 total) we would achieve 80% power to detect a difference on the Likert-scale of 0.5 (effect size) with a standard deviation of 1.0 between intervention and control groups. We applied the effect size and standard deviation from previous studies on GRADE SoF tables.[10–13] We assumed that 15% of those starting would not complete the survey, but we did not factor stratifying participants by stakeholder group into the calculation, as the aim of stratification was to balance participants rather than be sufficiently powered to detect subgroup effects.

Randomization

Participant response to a question on their role as a participant in this study will be used for stratification into one of four categories described in Figure 1. Participants within each of these categories will be randomly assigned in a 1:1 ratio to the WHO eTB or WHO TB arms.

Participants will also be randomly assigned to access one of two recommendations in a 1:1 ratio. The same two recommendations will be presented for both arms and selected because they contain mostly plain language and were more accessible to non-clinical participants.

Allocation Concealment

The allocation sequence is concealed using SurveyMonkey® software based on a commercial, but unknown algorithm without a pre-identified sequence.

Blinding

Participants will not be aware of their random allocation to WHO eTB or WHO TB until disclosure (see Figure 1). Thus, participants will be blinded for all outcomes except the secondary outcome of preference. Neutral language is used in both trial arms to prevent promotion of the intervention or comparison. Nevertheless, some participants may have accessed WHO TB recommendations in the past and a small number may have been aware of the development of WHO eTB.

Consultation and Pilot Testing

We will perform a pilot test for survey length, question relevance, and question clarity. Participants will be researchers and information technology developers affiliated with McMaster University. Revisions will be made to the questionnaire using pilot test feedback. Pilot test participants will not be included in the final analysis.

Statistical Analysis

We will conduct the analyses using IBM SPSS® (Statistical Package for Social Sciences) version 23.

Descriptive Analysis

We will summarize participant baseline characteristics and outcomes using means and standard deviations (SD) for continuous variables, and proportions for categorical variables.

Inferential Analysis

We will perform a primary analysis including all randomised participants, except for those who completed the survey in less than five minutes. We determined this cut-off a priori because user testing determined that it would be impossible to complete the work in that time. We will conduct a second per-protocol analysis which excludes participants who were flagged by SurveyMonkey® software (see below). For the outcomes of accessibility and satisfaction, we will use t-tests and mean differences 95% confidence intervals (95% CIs) to compare means and standard deviations (SDs) between the intervention and control groups. For the outcome of understanding, we will use χ^2 tests and risk difference (RD) with 95% CIs to compare the proportion of correct responses between groups. Finally, for preference, we will present preference as mean (SD) overall and for both trial arms. Skewness, Shapiro-Wilk tests, and histograms will be used to evaluate whether the distribution was shifted toward the same preference in both groups. Levene's test of equal variances will be used for all t-tests and degrees of freedom were adjusted for $p < 0.05$. We will report all p-values to three decimal places, with values less than 0.001 reported as < 0.001.

Dropouts, missing data, and poor-quality responses

We will use available case analysis for data from participants who responded to some survey questions after being assigned to the intervention. In order to prevent dropouts and missing data, we plan to implement the following strategies: (1) disseminating short emails to target stakeholder groups with a direct link to the survey, (2) informing participants that the survey will require 15 minutes, (3) participants who complete the survey have the option to enter a draw for a gift card, (4) all outcome questions are marked as mandatory, and (5) participants are randomised only after the collection of baseline characteristics.

We define participants who spend less than five minutes on the survey as inappropriate in our analyses, with the rationale that practical comprehension and completion of the survey could not be performed in five minutes. The average survey completion time is estimated to be 15 minutes. This is anticipated to prevent analysis of erroneous responses from participants who sought to simply gain access to the content of the survey or enter the gift card draw. For the per-protocol analysis, we will use a pre-defined but unknown SurveyMonkey® algorithm which flags poor-quality responses for straight-lining. Straight-lining is defined by SurveyMonkey® as responses to questions with the same answer option or pattern. Participants flagged for straight-lining, as well as those who spend less than five minutes on the survey, will be removed from the per-protocol analysis.

Interim Analysis

We will perform pre-planned interim analysis based on the thesis defence date of the first author (MM). The interim analysis will not be used to stop the study or draw final conclusions.

Discussion

Research in Context

To our knowledge, this is the first RCT comparing stakeholder feedback on the presentation of two guideline platforms. Nevertheless, several studies have explored stakeholder perceptions of guideline development and presentation, as well as the factors that influence their uptake. One qualitative study by Fearns, et al.[14] explored public perceptions of clinical practice guidelines and found that participants desired information to help them make decisions, but current numerical formats may not always be accessible to a public audience. Additionally, a content analysis by Santesso, et al.[15] found that patient versions of guidelines may not always address stakeholder needs, as they rarely include important EtD information, such as beliefs, values and preferences, accessibility, costs and feasibility. Furthermore, a realist review by Kastiner, et al.[16] which sought to identify the factors associated with guideline uptake, found that effective communication of content, including simple, clear and persuasive language, improved the implementability of guidelines by stakeholders.

This trial will expand on the methods of previous RCTs used to evaluate the presentation of guideline information, specifically, comparing new GRADE SoF tables to conventional formats. These studies evaluated participant understanding, accessibility, satisfaction and preference.[10,12,13] Carrasco-Labra, et al.[10] and Vandvik, et al.[13] identified that stakeholders preferred the presentation of risk differences over absolute risk estimates, as well as the inclusion of narrative statements to supplement numerical data. Furthermore, Akl, et al.[17] found that participants demonstrated a better understanding of strength of recommendations and quality of evidence when this information was presented as symbols, rather than numbers. Similar to our trial, these studies objectively evaluate perceptions of new to conventional formats to identify areas of improvement.

Strengths and Limitations

This study is anticipated to have several strengths. First, we use a randomised design conducted and reported in accordance with the CONSORT statement on randomised trials, which reduces the risk of confounding, selection and reporting bias.[9] Second, we use several previously validated outcomes from similar trials.[10–13] Third, we gather feedback from diverse group of stakeholders, thus improving the generalizability of findings using a carefully developed approach to presenting recommendations.[6]

This study also has some limitations. First, the ability to blind participants is limited, as some may have been aware of eTB development. We consider this probability to be small, as the main publication and awareness campaigns will begin after the majority of participants have been recruited. Second, data will be collected using an online survey and, thus, there is limited control over the environment in which the survey is performed. Third, participants may claim to

be part of more than one stakeholder group (e.g. a healthcare provider involved in research), but they are required to select just one for stratification. Fourth, we did not power our trial to conclusively evaluate results by participant strata.

Implications for Policy and Practice

Tuberculosis guideline recommendations developed by the WHO assist stakeholders in making evidence-informed decisions on TB prevention, diagnosis, treatment, and care.[2,3] According to the Guidelines International Network (G-I-N) and the National Institute for Health and Care Excellence (NICE), stakeholder engagement is important to ensuring that guideline products are feasible and acceptable to end-users.[18,19] This study will engage stakeholders in evaluating the presentation of recommendations through the outcomes of accessibility, understanding, and satisfaction, which are surrogates for the correct implementation of evidence in practice.

Implications for Research

This study is anticipated to demonstrate how RCTs may be used to compare stakeholder feedback on guideline platforms. Future studies should seek to explain the findings of this trial through qualitative and user testing techniques, such as the study by Rosenbaum, et al. for SoF tables.[11] Furthermore, additional trials should focus on specific stakeholder groups, such as patients and the public, to determine optimal ways to present recommendations.

S1 File. Trial Protocol.

References

1. World Health Organization. *Global Tuberculosis Report 2020*.; 2020.

2. World Health Organization. WHO | WHO End TB Strategy. WHO. Published 2015. Accessed September 16, 2020. http://www.who.int/tb/post2015_strategy/en/

3. Falzon D, Schünemann HJ, Harausz E, et al. World Health Organization treatment guidelines for drug-resistant tuberculosis, 2016 update. *Eur Respir J*. 2017;49(3). doi:10.1183/13993003.02308-2016

4. Moberg J, Oxman AD, Rosenbaum S, et al. The GRADE Evidence to Decision (EtD) framework for health system and public health decisions. *Health Res Policy Syst*. 2018;16(1):45. doi:10.1186/s12961-018-0320-2

5. Alonso-Coello P, Oxman AD, Moberg J, et al. GRADE Evidence to Decision (EtD) frameworks: a systematic and transparent approach to making well informed healthcare choices. 2: Clinical practice guidelines. *BMJ*. 2016;353. doi:10.1136/bmj.i2089

6. Hajizadeh A, Lotfi T, Falzon D, et al. Recommendation mapping of the World Health Organization's guidelines on tuberculosis: A new approach to digitizing and presenting recommendations. *J Clin Epidemiol*. 2021;0(0). doi:10.1016/j.jclinepi.2021.02.009

7. World Health Organization. eTB Guidelines: Database of recommendations for TB prevention and care. Accessed September 16, 2020. https://tuberculosis.evidenceprime.com/

8. Schünemann HJ, Wiercioch W, Brozek J, et al. GRADE Evidence to Decision (EtD) frameworks for adoption, adaptation, and de novo development of trustworthy recommendations: GRADE-ADOLOPMENT. *J Clin Epidemiol*. 2017;81:101-110. doi:10.1016/j.jclinepi.2016.09.009

9. Moher D, Hopewell S, Schulz KF, et al. CONSORT 2010 Explanation and Elaboration: updated guidelines for reporting parallel group randomised trials. *BMJ*. 2010;340. doi:10.1136/bmj.c869

10. Carrasco-Labra A, Brignardello-Petersen R, Santesso N, et al. Comparison between the standard and a new alternative format of the Summary-of-Findings tables in Cochrane review users: study protocol for a randomized controlled trial. *Trials*. 2015;16(1):164. doi:10.1186/s13063-015-0649-6

11. Rosenbaum SE, Glenton C, Nylund HK, Oxman AD. User testing and stakeholder feedback contributed to the development of understandable and useful Summary of Findings tables for Cochrane reviews. *J Clin Epidemiol*. 2010;63(6):607-619. doi:10.1016/j.jclinepi.2009.12.013

12. Yepes-Nuñez JJ, Morgan RL, Mbuagbaw L, et al. Two alternatives versus the standard Grading of Recommendations Assessment, Development and Evaluation (GRADE) summary of

findings (SoF) tables to improve understanding in the presentation of systematic review results: a three-arm, randomised, controlled, non-inferiority trial. *BMJ Open*. 2018;8(1):e015623. doi:10.1136/bmjopen-2016-015623

13. Vandvik PO, Santesso N, Akl EA, et al. Formatting modifications in GRADE evidence profiles improved guideline panelists comprehension and accessibility to information. A randomized trial. *J Clin Epidemiol*. 2012;65(7):748-755. doi:10.1016/j.jclinepi.2011.11.013

14. Fearns N, Kelly J, Callaghan M, et al. What do patients and the public know about clinical practice guidelines and what do they want from them? A qualitative study. *BMC Health Serv Res*. 2016;16. doi:10.1186/s12913-016-1319-4

15. Santesso N, Morgano GP, Jack SM, et al. Dissemination of Clinical Practice Guidelines: A Content Analysis of Patient Versions. *Med Decis Mak Int J Soc Med Decis Mak*. 2016;36(6):692-702. doi:10.1177/0272989X16644427

16. Kastner M, Bhattacharyya O, Hayden L, et al. Guideline uptake is influenced by six implementability domains for creating and communicating guidelines: a realist review. *J Clin Epidemiol*. 2015;68(5):498-509. doi:10.1016/j.jclinepi.2014.12.013

17. Akl EA, Maroun N, Guyatt G, et al. Symbols were superior to numbers for presenting strength of recommendations to health care consumers: a randomized trial. *J Clin Epidemiol*. 2007;60(12):1298-1305. doi:10.1016/j.jclinepi.2007.03.011

18. Guidelines International Network. G-I-N PUBLIC Toolkit: Patient and Public Involvement in Guidelines — Guidelines International Network. Accessed September 16, 2020. https://g-i-n.net/working-groups/gin-public/toolkit

19. National Institute for Health and Care Excellence. A report on a study to evaluate patient/carer membership of the first NICE Guideline Development Groups. :34.
