# Supplementary material for: Comparing the usability of the World Health Organization’s conventional tuberculosis guidelines to the eTB recommendations map: A two-arm superiority randomised controlled trial
Source: PLOS Glob Public Health. 2022 Oct 14;2(10):e0001166. doi: 10.1371/journal.pgph.0001166 (PMC10021182; doi:10.1371/journal.pgph.0001166)
Supplement: S2 File — (PDF) [file pgph.0001166.s003.pdf]

S2 File. Consent, survey, and recruitment details.

Survey consent form

Study Name: Exploring Stakeholder Perceptions of the World Health Organization's Tuberculosis Guidelines

Purpose: Thank you for your interest in this survey! We would like to explore your perceptions of the presentation of guidelines and recommendations offered by the World Health Organization (WHO) Global Tuberculosis Department. Your feedback will help us learn more about the WHO tuberculosis (TB) recommendation presentation and engagement process. This information will be used to improve the presentation of WHO TB recommendations.

This survey will take approximately 15 minutes to complete.
As a thank you, you will be invited to enter a draw for one of five $50 Amazon dift cards at the end.

Confidentiality: This survey is anonymous and all information will remain confidential. Survey responses will be secured on a password protected device only to be accessed by the research team. No personally identifiable information will be shared with individuals or organizations outside of the research team, including the WHO. We will only collect personal identifying information (your name and email) at the end of the survey if you agree to be contacted for follow-up questions.

Risks: We do not foresee any risks from your participation.

Voluntary Participation: Your participation in this survey is voluntary. You are free to leave the survey at any time. If you choose to leave, we will only be able to remove your responses if you have provided us with your name and email. Otherwise, the anonymous information cannot be removed.

Ethics Approval: This study has been reviewed by the Hamilton Integrated Research Ethics Board (HiREB). If you have any questions about your rights as a research participant, please call the Office of the Chair, HiREB at 1-905-521-2100 x 42013

Researcher Contacts: Micayla Matthews matthm9@mcmaster.ca; Holger Schünemann, schuneh@mcmaster.ca, McMaster University

Consent: By participating in this study you do not give up any rights to which you may be entitled under the law. Your filling out of the survey provides consent for participation in this study. Thank you in advance for your participation.

S2 File. Consent, survey, and recruitment details.

Survey questions

Demographics: To better understand your perspective, we will begin by asking for a few anonymous questions about yourself.

1. In what role(s) are you participating in this survey? Check all that apply.
   - a. Patient
   - b. Caregiver
   - c. Member of the public
   - d. Patient advocate or patient group representative
   - e. Healthcare employer
   - f. Guideline developer
   - g. Member of a professional association
   - h. Peer review editor (for journals or guidelines)
   - i. Journalist
   - j. Other
     - i. Please specify ___________
2. In which setting(s) do you currently work or live?
   - a. Low and middle income country (LMIC)
   - b. High income country (HIC)
   - c. Both LMIC and HIC
   - d. Prefer not to respond

Please list which country (or countries) you currently work or live. _________

3. What is the highest level of school you have completed?
   - ~~a.~~ Some primary or secondary school
   - b. High school, diploma or the equivalent (for example: GED)
   - ~~c.~~ Certificate or college diploma
   - d. Bachelor’s degree
   - e. Professional degree (e.g. MD, DDS, JD)
   - ~~f.~~ Graduate Degree (e.g. Masters, PhD)
   - ~~g.~~ Both professional and graduate degrees
   - h. Other (please specify) ______
   - i. Prefer not to respond
4. How ~~long~~ many years have you been involved in ~~TB~~ tuberculosis-focused work?
   - a. <1 year
   - b. 1-2 years
   - c. 3-5 years
   - d. 6-9 years
   - e. >10 years
   - f. Not applicable (e.g. I am a patient)
   - g. Prefer not to respond
5. What is your age?
   - a. < 25
   - b. 26-35
   - c. 36-45
   - d. 46-55
   - e. 56-65
   - f. 66-75
   - g. 76 <
   - h. Prefer not to respond
6. What is your gender?
   - a. Female
   - b. Male
   - c. Other
   - d. Prefer not to respond

Accessing WHO TB Guidelines

7. In what role are you most likely to access WHO tuberculosis guidelines? Select the one category that describes you best.
    a. Patient, Public, Caregiver, Patient Advocate/Representative, or Journalist
    b. Healthcare Provider or Employer
    c. Policymaker, Guideline Developer, Insurer, Department of Health Representative, Program Manager, or Manufacturer
    d. Academic, Researcher, Funder, or Peer Review Editor
8. Have you ever accessed any tuberculosis guidelines, recommendations, or policy advice in the past?
    a. Yes
    b. No
    c. Unsure
9. Do you plan on accessing any tuberculosis guidelines, recommendations, or policy advice in the future?
    a. Yes
    b. No
    c. Unsure
10. Specifically, have you ever accessed WHO tuberculosis guidelines, recommendations, or policy advice?
    a. Yes
    b. No
    c. Unsure
11. How comfortable are you with basic information and communication technologies? (e.g. internet search, smartphone, email)
    a. Very uncomfortable
    b. Uncomfortable
    c. Somewhat uncomfortable
    d. Neutral
    e. Somewhat comfortable
    f. Comfortable
    g. Very comfortable

WHO Tuberculosis Guidelines Website/WHO eTB Guidelines
We are interested in your perspective on the WHO Tuberculosis guidelines website/WHO eTB guidelines available [at this link]. Please explore the website and follow the short instructions below to access a recommendation.

12. You seek to determine whether a centralized or decentralized model of care is recommended for patients with multidrug tuberculosis (MDR-TB)/You seek to determine whether latent tuberculosis infection (LTBI) testing and treatment should be considered for prisoners.

- Use the search functions to browse Tuberculosis (TB) publications
- Locate the most recent guideline on drug resistant tuberculosis
- Open the document and search for the recommendation

13. What is the recommendation strength?
    a. Strong recommendation for the intervention
    b. Conditional recommendation for the intervention
    c. Strong recommendation against the intervention
    d. Conditional recommendation against the intervention
    e. Recommendation not found
14. What is the certainty of evidence?
    a. Moderate
    b. Low
    c. Very ow
    d. Low to very low
    e. Recommendation not found
15. On which page does the evidence to decision (EtD) table for this recommendation start?
    a. Page 16

b. Page 5
c. Page 82
d. Page 2
e. EtD not found

16. How satisfied are you with the presentation of the home page?
    a. Very dissatisfied
    b. Dissatisfied
    c. Somewhat dissatisfied
    d. Neutral
    e. Somewhat satisfied
    f. Satisfied
    g. Very Satisfied
    h. Please share what you like or dislike (optional)
17. How satisfied are you with the presentation of the list of recommendations?
    a. Very dissatisfied
    b. Dissatisfied
    c. Somewhat dissatisfied
    d. Neutral
    e. Somewhat satisfied
    f. Satisfied
    g. Very Satisfied
    h. Please share what you like or dislike (optional)
18. How satisfied are you with the presentation of this individual recommendation?
    a. Very dissatisfied
    b. Dissatisfied
    c. Somewhat dissatisfied
    d. Neutral
    e. Somewhat satisfied
    f. Satisfied
    g. Very Satisfied
    h. Please share what you like or dislike (optional)
19. Please answer the following questions on your experience with the accessibility of the WHO Tuberculosis Guidelines website.

    a. The website was easy to navigate.
        i. Strongly agree
        ii. Agree
        iii. Somewhat agree
        iv. Neutral
        v. Somewhat disagree
        vi. Disagree
        vii. Strongly disagree

    b. It is easy to find the information
        i. Strongly agree
        ii. Agree
        iii. Somewhat agree
        iv. Neutral
        v. Somewhat disagree
        vi. Disagree
        vii. Strongly disagree

    c. It was easy to understand the information
        i. Strongly agree
        ii. Agree
        iii. Somewhat agree

iv. Neutral
v. Somewhat disagree
vi. Disagree
vii. Strongly disagree

d. The information was presented in a way that would help me make a decision
   i. Strongly agree
   ii. Agree
   iii. Somewhat agree
   iv. Neutral
   v. Somewhat disagree
   vi. Disagree
   vii. Strongly disagree

20. Please provide any final comments on your experience with the WHO Tuberculosis Guidelines Website/WHO eTB Guidelines website, including how to make it more accessible or relevant to you (optional).
    a. ____________

Preference: The purpose of this project is to compare the accessibility of recommendations from the current WHO Tuberculosis Guidelines website, to an alternative WHO eTB Guidelines website.

- Click here for a short demonstration of the current WHO Tuberculosis guidelines website
- Click here for a short demonstration of the alternative WHO eTB Guidelines website

Between the WHO Tuberculosis Guidelines (current website), and the WHO eTB guidelines (alternative website), which do you prefer?

a. Strongly prefer WHO Tuberculosis Guidelines
b. Prefer WHO Tuberculosis Guidelines
c. Somewhat Prefer WHO Tuberculosis Guidelines
d. Same Preference for WHO Tuberculosis and eTB Guidelines
e. Somewhat Prefer WHO eTB Guidelines
f. Prefer WHO eTB Guidelines
g. Strongly Prefer WHO eTB Guidelines

What additional resources would you like to see as part of WHO Tuberculosis Guidelines in the future? Check all that apply.

a. Plain language summaries of evidence and recommendations
b. Decision aids (example: flowcharts, decision scoring system, etc.)
c. Implementation tools (example: WHO ENGAGE-TB manual to promote community engagement)
d. Translation to other languages
e. Unsure
f. Other resources (please specify) ______

This is the end of the survey. Thank you for participating in this project!

S2 File. Consent, survey, and recruitment details.

Description of understanding instructions, questions, and answer key

	Guideline Reference	Recommenda-tion Statement	Survey Instructions	Understanding Questions	Answer Key
**WHO eTB**	WHO consolidated guidelines on tuberculosis. Module 4: Treatment. Drug-resistant tuberculosis treatment. World Health Organization, 2020. License: CC BY-NC-SA 3.0 IGO.	A decentralized model of care is recommended over a centralized model for patients on MDR-TB treatment.	You seek to determine whether a centralized or decentralized model of care is recommended for patients with multidrug resistant tuberculosis (MDR-TB).<br>• Use the search function to find this recommendation.<br>• Click on the recommendation.	1. What is the recommendation strength?<br>2. What is the certainty of evidence?<br>3. On which page does the evidence to decision (EtD) table for this recommendation start?	1. Conditional recommendation for the intervention<br>2. Very low<br>3. 82 (Guidelines for treatment of drug-susceptible tuberculosis and patient care 2017 update, Annex 4, PICO 11)
**WHO TB**	WHO consolidated guidelines on tuberculosis. Module 4: Treatment. Drug-resistant tuberculosis treatment. World Health Organization, 2020. License: CC BY-NC-SA 3.0 IGO.	A decentralized model of care is recommended over a centralized model for patients on MDR-TB treatment.	You seek to determine whether a centralized or decentralized model of care is recommended for patients with multidrug resistant tuberculosis (MDR-TB).<br>• Use the search function to browse Tuberculosis (TB) publications.<br>• Locate the most recent guideline on drug resistant tuberculosis.<br>• Open the document and search for the recommendation.	1. What is the recommendation strength?<br>2. What is the certainty of evidence?<br>3. On which page does the evidence to decision (EtD) table for this recommendation start?	1. Conditional recommendation for the intervention<br>2. Very low<br>3. 82
**WHO eTB**	WHO consolidated guidelines on tuberculosis. Module 1: Prevention. Tuberculosis preventative treatment. World Health Organization, 2020. License: CC BY-NC-SA 3.0 IGO.	Systematic LTBI testing and treatment may be considered for prisoners, health workers, immigrants from other countries with a high TB burden, homeless people and people who use drugs.	You seek to determine whether latent tuberculosis infection (LTBI) testing and treatment should be considered for prisoners.<br>• Use the search function to find this recommendation.<br>• Click on the recommendation.	1. What is the recommendation strength?<br>2. What is the certainty of evidence?<br>3. On which page does the evidence to decision (EtD) table for this recommendation start?	1. Conditional recommendation for the intervention<br>2. Low to very low<br>3. 5
**WHO TB**	WHO consolidated guidelines on tuberculosis. Module 1: Prevention. Tuberculosis preventative treatment. World Health Organization, 2020. License: CC	Systematic LTBI testing and treatment may be considered for prisoners, health workers, immigrants from other countries with a high TB burden, homeless people and people who use drugs.	You seek to determine whether latent tuberculosis infection (LTBI) testing and treatment should be considered for prisoners.<br>• Use the search function to browse Tuberculosis (TB) publications.	1. What is the recommendation strength?<br>2. What is the certainty of evidence?<br>3. On which page does the evidence to decision (EtD) table for this	1. Conditional recommendation for the intervention<br>2. Low to very low<br>3. 5

	BY-NC-SA 3.0 IGO.		• Locate the most recent guideline on tuberculosis treatment.<br>• Open the document and search for the recommendation.	recommendation start?	

S2 File. Consent, survey, and recruitment details.

Preference outcome demonstrations

Accessing Tuberculosis Recommendations using the **WHO eTB Guidelines** Website
Short Demonstration

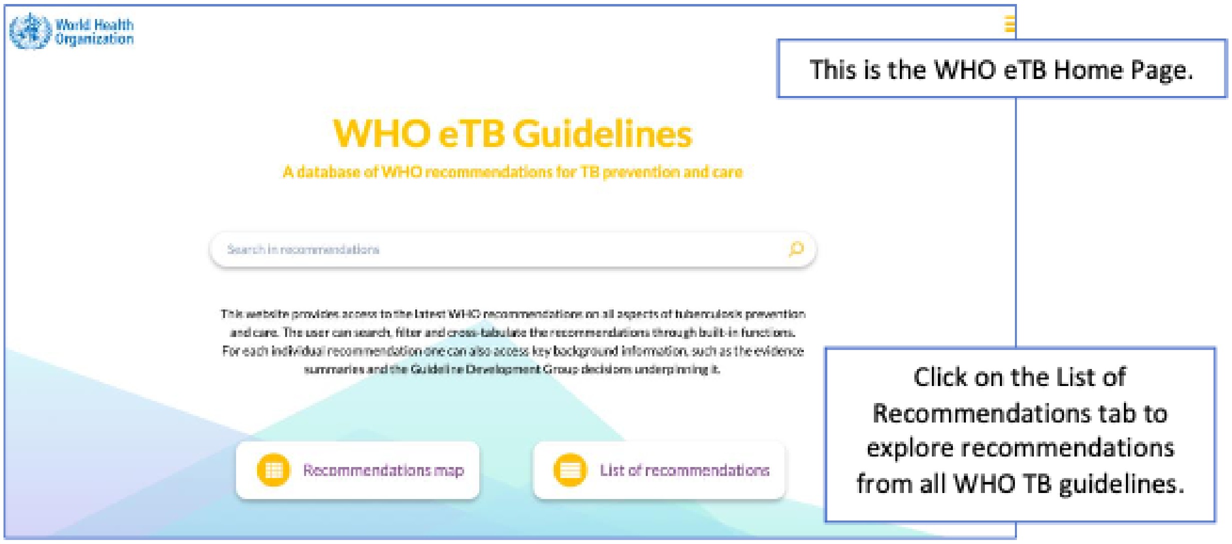

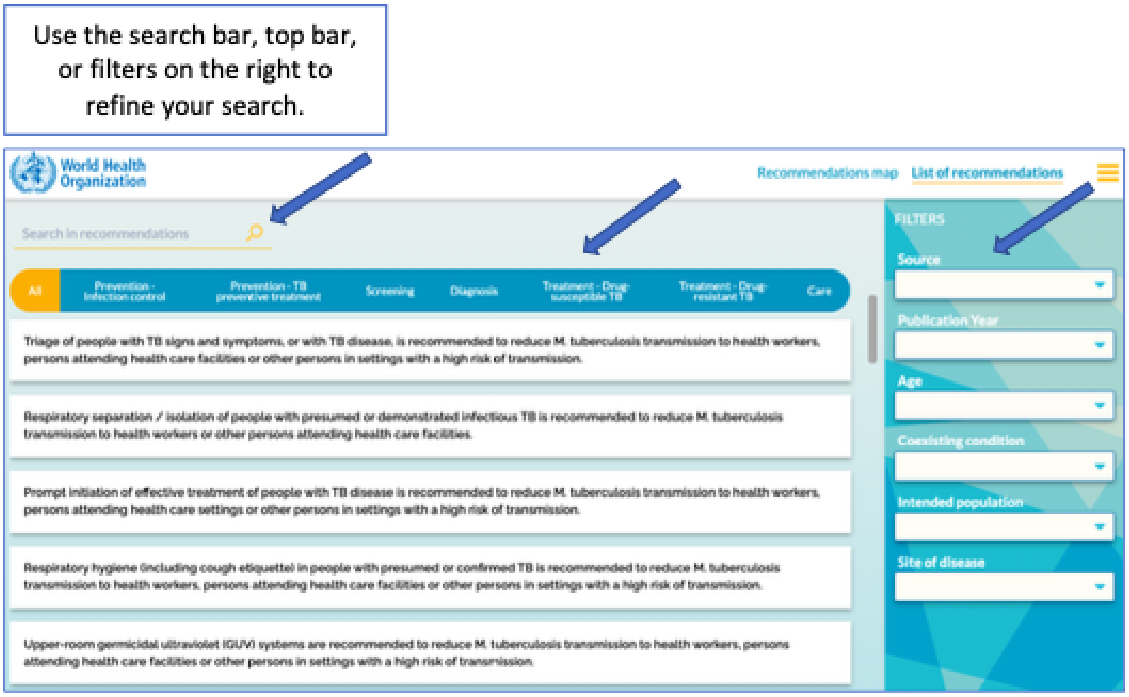

S2 File. Consent, survey, and recruitment details.

Click on a recommendation to see this page.

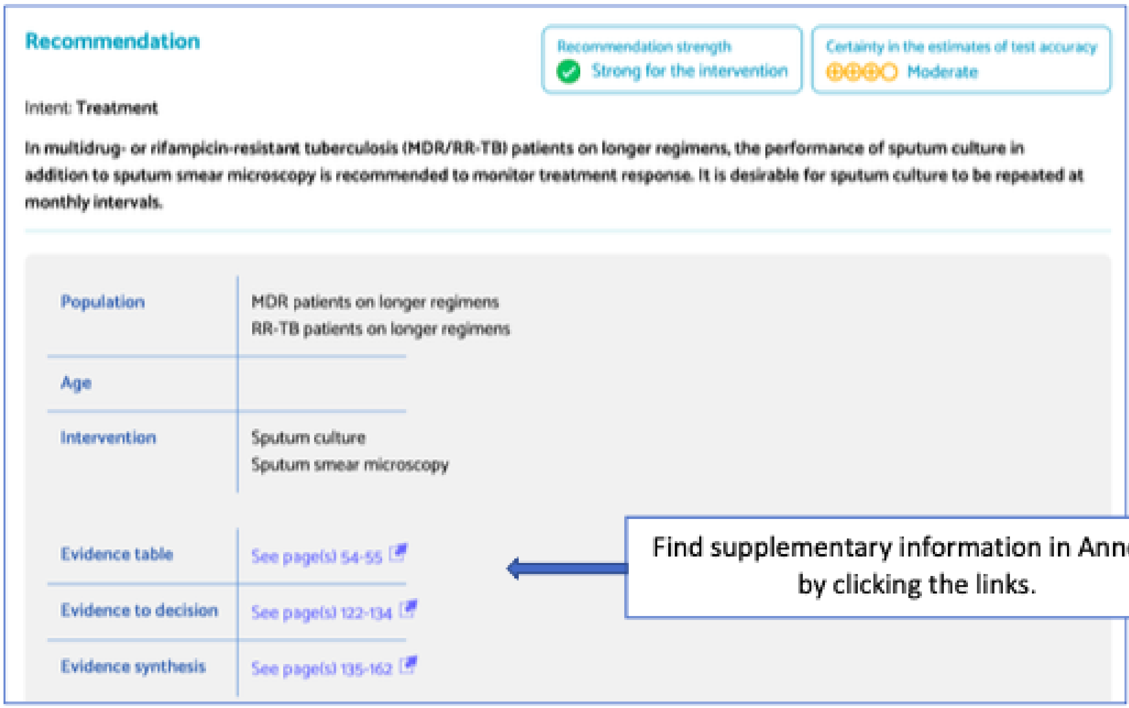

Find supplementary information in Annexes by clicking the links.

Click on the recommendations map to see all WHO TB recommendations organized by their population and intervention.

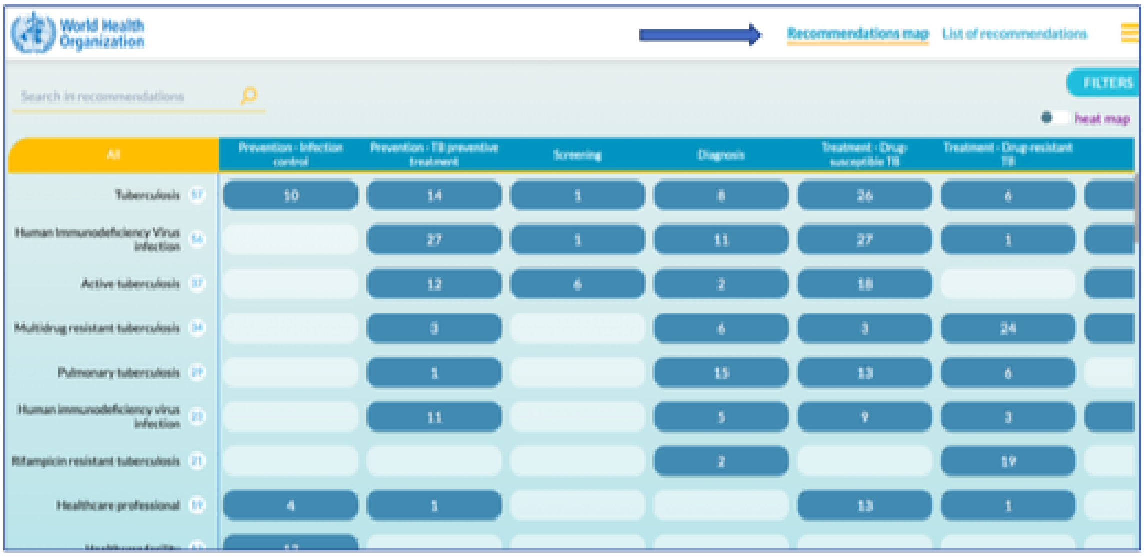

S2 File. Consent, survey, and recruitment details.

Accessing TB Recommendations using the **WHO Tuberculosis Guidelines** Website
Short Demonstration

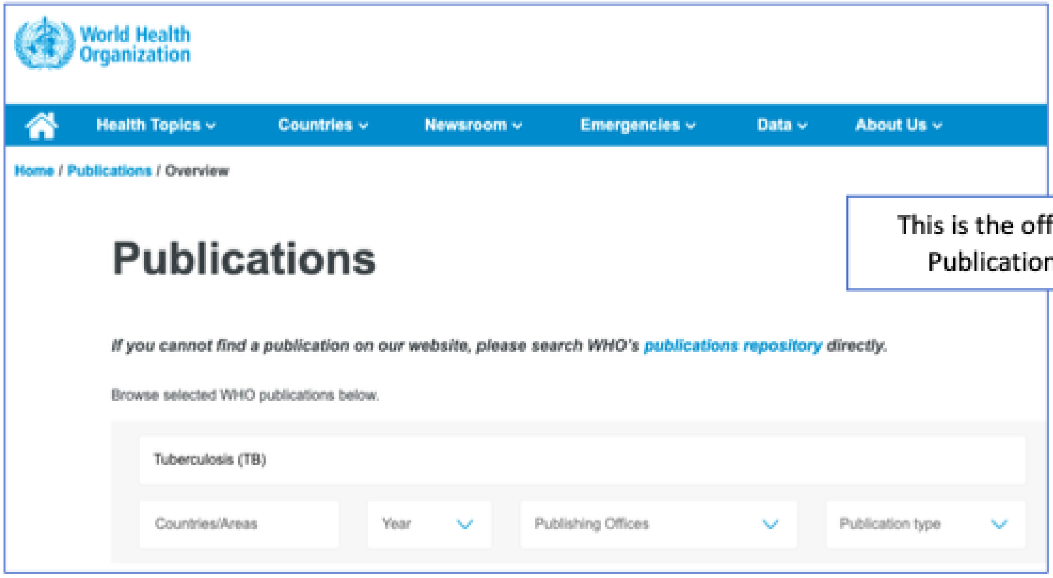

This is the official WHO Publications Page.

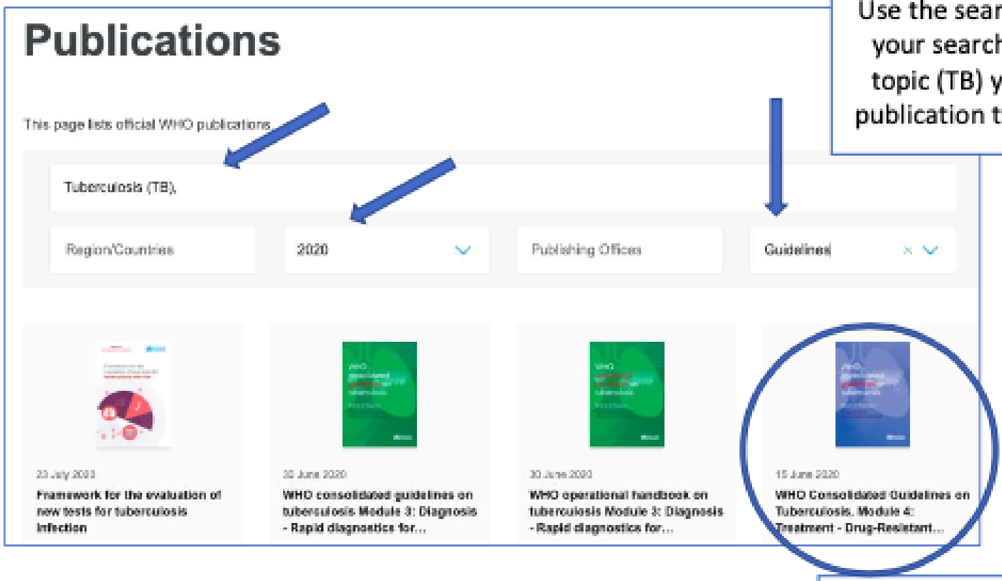

Use the search bar to specify your search such as health topic (TB) year (2020), and publication type (Guidelines).

Select and download the Guideline PDF of your choice.

S2 File. Consent, survey, and recruitment details.

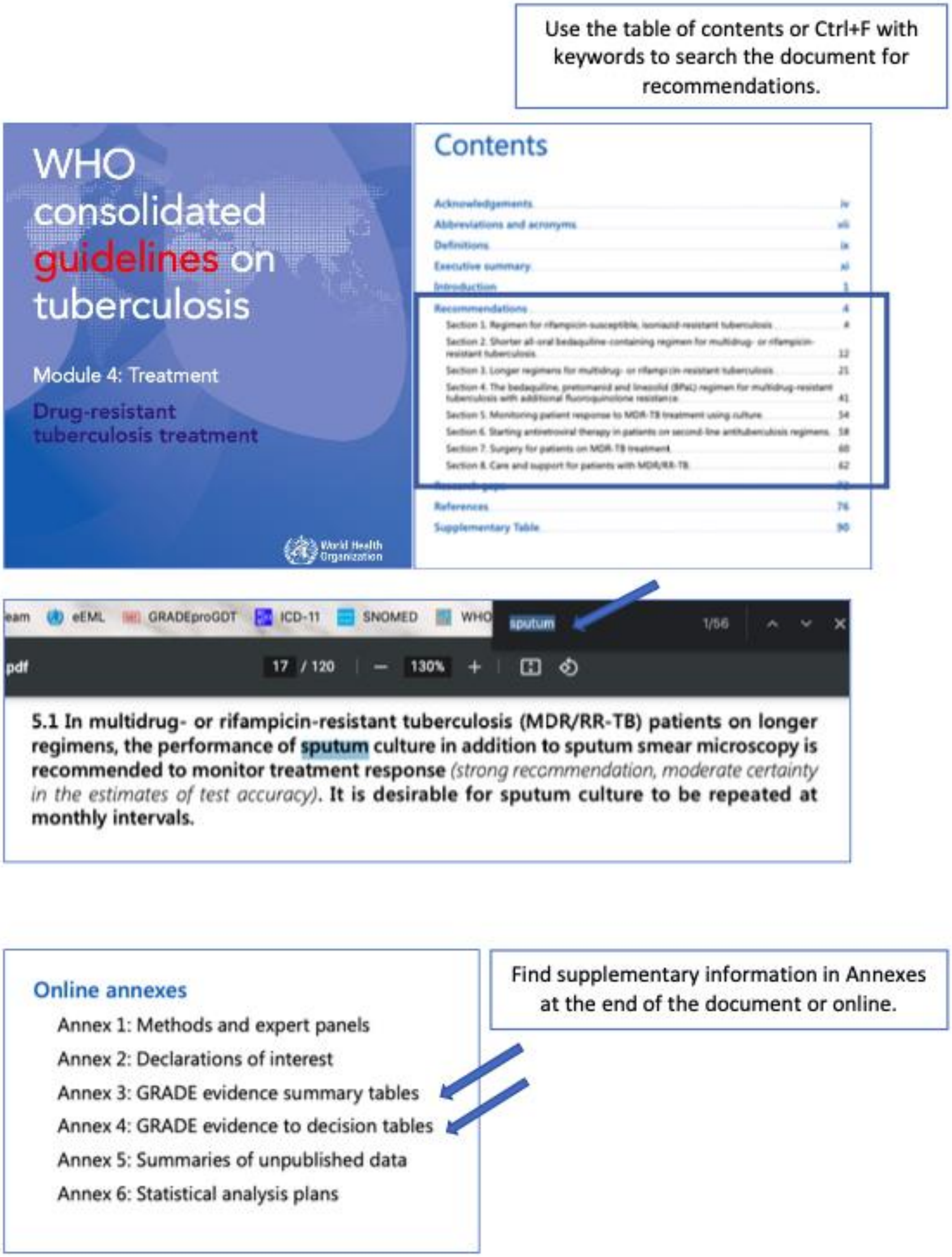

S2 File. Consent, survey, and recruitment details.

Stakeholder identification and participant recruitment

Stakeholder identification

10 Ps Framework for Stakeholder Engagement[1]	
**Patients, the Public, Caregivers and Patient Advocates**	Consumers of healthcare, families, consumer advocacy organizations
**Providers of Healthcare**	Healthcare professionals (physicians, nurses, etc.) and health centers or community organizations that provide care to patients and populations
**Purchasers**	Employers, the self-insured, government, and other entities responsible for underwriting to costs of healthcare
**Payers of Health Services**	Insurers, insurance exchanges, individuals with deductibles, others responsible for reimbursement
**Policymakers**	Government, departments of health, professional associations, intermediaries, other policymaking groups
**Program Managers**	Member countries, organizations
**Product Makers**	Drug and device manufacturers
**Principal Investigators and their Research Teams**	Researchers, academics and their funders
**Peer Review Editors**	Journals, guidelines

Email participant recruitment

In this targeted snowball recruitment strategy, potential participants contacted with a request to participate and share the survey within their networks, including people from any background who are users or potential users of TB guidelines, recommendations, and policy advice.

	Outreach Dates	No. Estimates
**WHO TB Guideline Development Group (GDG) Members**	February - March, 2021	140
**Pai Global TB Group and TB Public Private Mix Learning Network**	March, 2021; June, 2021	1500
**PubMed targeted searches of authors**	March, 2021	50
**Canadian infectious disease and Indigenous groups**	April, 2021	85
**Public relations and social media officials from organizations including:** The Union, ECDC TB, Stop TB partnership, TB Alliance, The Global Fund, CDC TB, FIRS, Global TB Caucus, REACH, Stop TB Canada, GIN Member, Cochrane Canada	May, 2021	Unknown
**WHO National TB Programme Managers**	June, 2021	240
**South African infectious disease and guideline groups**	June, 2021	Unknown

Social media (Twitter and LinkedIn) participant recruitment

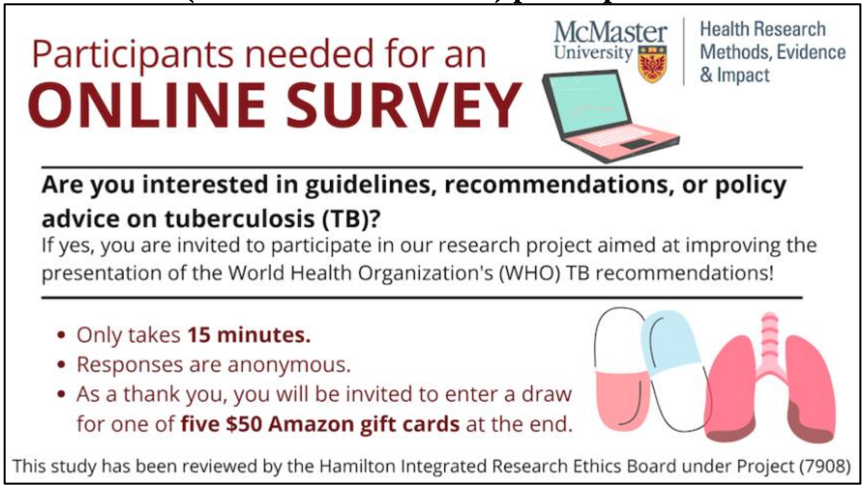

Reference

1. Concannon T, Meissner P, Grunbaum J, et al. A new taxonomy for stakeholder engagement in patient-centered outcomes research. *Journal of general internal medicine*. doi:10.1007/s11606-012-2037-1
