# Supplementary material for: Comparing the usability of the World Health Organization’s conventional tuberculosis guidelines to the eTB recommendations map: A two-arm superiority randomised controlled trial
Source: PLOS Glob Public Health. 2022 Oct 14;2(10):e0001166. doi: 10.1371/journal.pgph.0001166 (PMC10021182; doi:10.1371/journal.pgph.0001166)
Supplement: S3 File — (PDF) [file pgph.0001166.s004.pdf]

S3 File. Interim and per-protocol analysis details.

Interim analysis

Figure 1. Flow of participants through the study (Interim analysis)

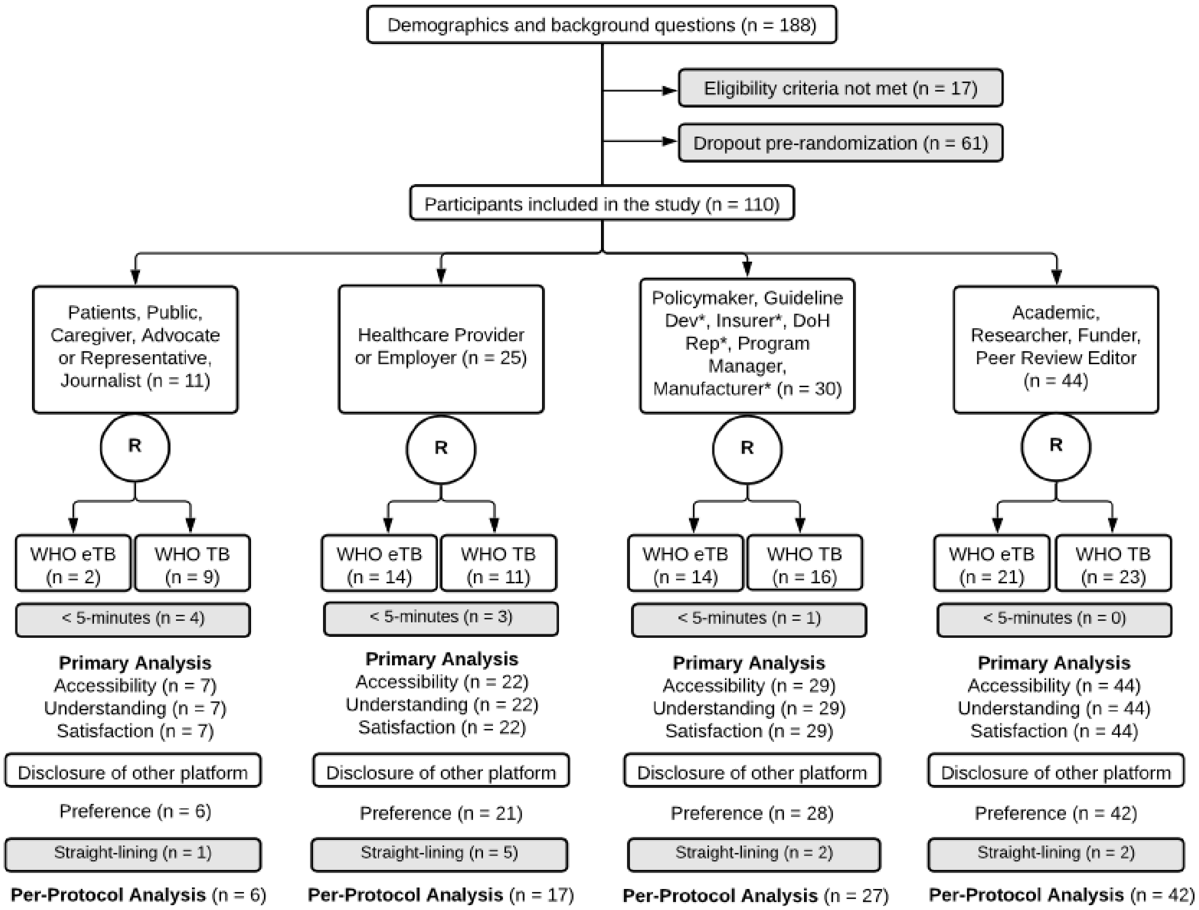

*Abbreviations: Guideline Dev: Guideline developer, Insurer: Insurer of health services, DoH Rep: Department of health representative, Manufacturer: Drug or device manufacturer

Table 1. Baseline characteristics of participants per group (Interim analysis)

	WHO eTB (n = 49)	WHO TB (n = 53)
Gender: n (%)		
Female	22 (45)	30 (56)
Male	27 (55)	21 (40)
Other	-	1 (2)
Prefer not to respond	-	1 (2)
Age (years): n (%)		
< 25	1 (2)	1 (2)
26-35	12 (23)	13 (22)
36-45	18 (35)	20 (34)
46-55	10 (20)	13 (22)
56-65	6 (12)	10 (17)
66-75	3 (6)	2 (3)
Prefer not to respond	1 (2)	-
Setting: n (%)		
HIC	11 (23)	18 (34)

S3 File. Interim and per-protocol analysis details.

LMIC<br>HIC and LMIC	29 (59)<br>9 (18)	30 (57)<br>5 (9)
Education: n (%)<br>Primary<br>College<br>Bachelor<br>Professional<br>Graduate<br>Professional and graduate	 -<br>1 (2)<br>4 (8)<br>6 (12)<br>23 (47)<br>15 (31)	 1 (2)<br>1 (2)<br>3 (6)<br>13 (24)<br>23 (43)<br>12 (23)
TB work (years): n (%)<br>< 1<br>1-2<br>3-5<br>6-9<br>> 10<br>Not applicable	 1 (2)<br>2 (4)<br>4 (8)<br>7 (14)<br>33 (68)<br>2 (4)	 2 (4)<br>3 (6)<br>6 (11)<br>4 (7)<br>38 (72)<br>-

Abbreviations: WHO, World Health Organization; TB, Tuberculosis; HIC, high income country; LMIC, low- and middle-income country

Table 2. Overall accessibility of information [mean (SD)]

**Domain**[a]	**WHO eTB (n = 49)**	**WHO TB (n = 53)**	**MD (95% CI)**	**p value**
It was easy to find the information.[a]	5.6 (1.3)	4.1 (1.8)	1.4 (0.8, 2.0)[b]	< 0.001
It was easy to understand the information.[a]	5.6 (1.1)	4.9 (1.6)	0.8 (0.3, 1.3)[b]	0.004
The information was presented in a way that would help me make a decision.[a]	5.7 (1.2)	4.9 (1.4)	0.8 (0.3, 1.3)	0.003
This website was easy to navigate.[a]	5.5 (1.5)	4.0 (1.8)	1.5 (0.9, 2.1)[b]	< 0.001
Overall Accessibility[c]	**5.6 (1.2)**	**4.5 (1.4)**	**1.1 (0.6, 1.6)[b]**	**< 0.001**

Abbreviations: SD, standard deviation; WHO, World Health Organization; TB, tuberculosis; MD, mean difference; CI, confidence interval

[a] Likert-scale from 1 = strongly disagree to 7 = strongly agree

[b] Equal variances could not be assumed using Levene's test, degrees of freedom adjusted

[c] Composite of four domains (primary outcome)

Table 3. Percentage (%) of participants who responded correctly to understanding questions

	**WHO eTB (n = 49)**	**WHO TB (n = 53)**	**Risk Difference (95% CI)**	**p value**[a]
What is the recommendation strength?	76	66	10 (-8, 28)	0.294
What is the certainty of evidence?	57	51	6 (-13, 25)	0.530
On which page does the evidence to decision (EtD) table for this recommendation start?	65	8	57 (43, 73)	< 0.001

[a] Pearson's chi-square

Table 4. Satisfaction with the presentation of platform pages [mean (SD)]

**Page**	**WHO eTB (n = 49)**	**WHO TB (n = 53)**	**MD (95% CI)**	**p value**
Home page[a]	5.7 (1.0)	4.1 (1.8)	1.7 (1.1, 2.3)[b]	< 0.001
List of recommendations[a]	5.6 (1.1)	5.2 (1.4)	0.4 (-0.1, 0.9)[b]	0.143
Individual recommendation[a]	5.8 (1.1)	5.1 (1.4)	0.7 (0.2, 1.2)[b]	0.011

Abbreviations: SD, standard deviation; WHO, World Health Organization; TB, tuberculosis; MD, mean difference; SE, standard error

[a] Likert-scale from 1 = very dissatisfied to 7 = very satisfied

[b] Equal variances could not be assumed using Levene's test, degrees of freedom adjusted

S3 File. Interim and per-protocol analysis details.

Preference

Overall, participants (n = 97), on average, "somewhat preferred WHO eTB" (4.9; SD 1.8), after reviewing demonstrations of both platforms. There was no statistically significant difference in mean preference between participants who were assigned to WHO eTB (5.0; SD 1.7), or WHO TB (4.7; SD 2.0) (p = 0.481). Both arms were left-skewed toward this preference (p < 0.001)

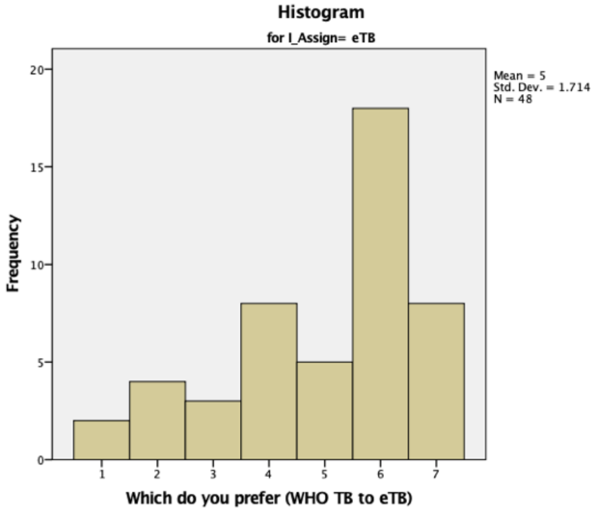

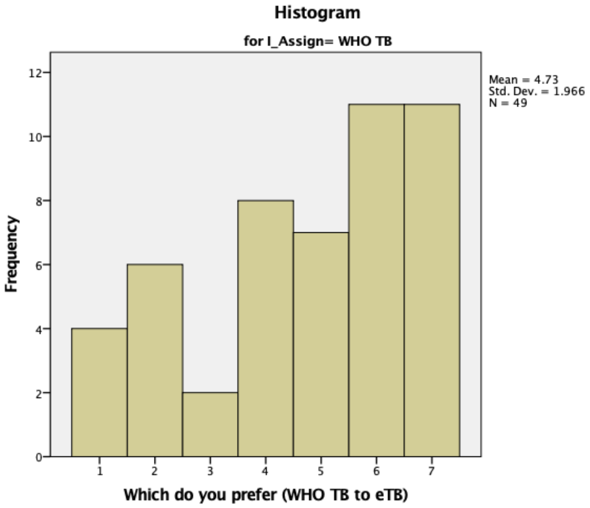

Table 5. Shapiro-Wilk test

Shapiro-Wilk	Statistic	Degrees of freedom	p value
WHO eTB (n = 48)	0.873	48	< 0.001
WHO TB (n = 49)	0.888	49	< 0.001

Table 6. Skewness test

Skewness	Statistic	Standard error
WHO eTB (n = 48)	-0.821	0.343
WHO TB (n = 49)	-0.556	0.340

S3 File. Interim and per-protocol analysis details.

Per-protocol analysis

Table 1. Overall accessibility of information [mean (SD)] (Per-Protocol Analysis)

Domain[a]	WHO eTB (n = 111)	WHO TB (n = 116)	MD (95% CI)[b]	p value
It was easy to find the information	5.5 (1.2)	4.4 (1.9)	1.1 (0.7, 1.5)	< 0.001
It was easy to understand the information	5.6 (1.0)	5.0 (1.6)	0.6 (0.2, 0.9)	0.001
The information was presented in a way that would help me make a decision	5.7 (1.0)	5.0 (1.5)	0.7 (0.3, 1.0)	< 0.001
This website was easy to navigate	5.6 (1.2)	4.3 (1.8)	1.3 (0.9, 1.7)	< 0.001
Overall Accessibility[c]	**5.6 (1.0)**	**4.7 (1.5)**	**0.9 (0.6, 1.3)**	**< 0.001**

Abbreviations: SD, standard deviation; WHO, World Health Organization; TB, tuberculosis; MD, mean difference; CI, confidence interval

[a] Likert-scale from 1 = strongly disagree to 7 = strongly agree

[b] Equal variances could not be assumed using Levene’s test, degrees of freedom adjusted

[c] Composite of four domains (primary outcome)

Table 2. Percentage (%) of participants who responded correctly to understanding questions (Per-Protocol Analysis)

Question	WHO eTB (n = 111)	WHO TB (n = 116)	Risk Difference (95% CI)	p value[a]
What is the recommendation strength?	72	53	19 (7, 31)	0.004
What is the certainty of evidence?	55	34	21 (8, 34)	0.001
On which page does the evidence to decision (EtD) table for this recommendation start?	56	8	48 (38, 58)	< 0.001

[a] Pearson’s chi-square

Table 3. Satisfaction with the presentation of platform pages [mean (SD)] (Per-Protocol Analysis)

Page[a]	WHO eTB (n = 111)	WHO TB (n = 116)	MD (95% CI)	p value
Home page	5.6 (1.0)	4.6 (1.8)	1.0 (0.6, 1.4)[b]	< 0.001
List of recommendations	5.5 (1.1)	5.3 (1.3)	0.2 (-0.1, 0.5)[b]	0.214
Individual recommendation	5.6 (1.1)	5.3 (1.3)	0.3 (-0.04, 0.6)	0.084

Abbreviations: SD, standard deviation; WHO, World Health Organization; TB, tuberculosis; MD, mean difference; SE, standard error

[a] Likert-scale from 1 = very dissatisfied to 7 = very satisfied

[b] Equal variances could not be assumed using Levene’s test, degrees of freedom adjusted

S3 File. Interim and per-protocol analysis details.

Additional details on preference outcome analysis

Overall, participants (n = 217), on average, “somewhat preferred WHO eTB” (4.8; SD 1.8), after reviewing demonstrations of both platforms. There was no statistically significant difference in mean preference between participants who were assigned to WHO eTB (5.0; SD 1.6), or WHO TB (4.6; SD 2.0) ($p = 0.091$). Both arms were left-skewed toward this preference ($p < 0.001$).

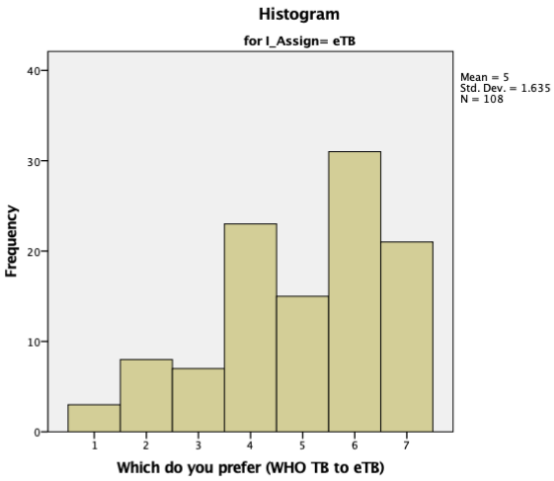

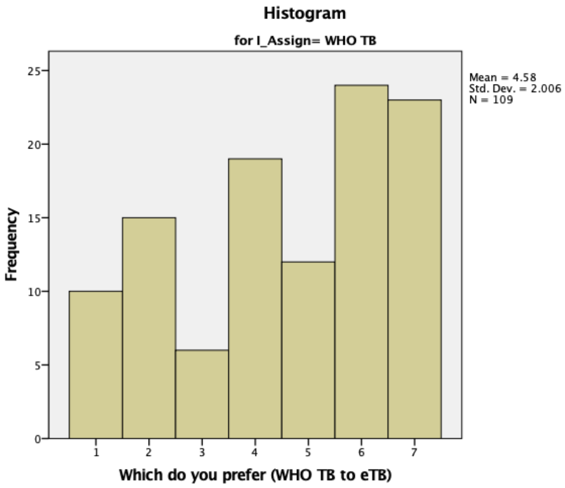

Table 4. Shapiro-Wilk test

Shapiro-Wilk	Statistic	Degrees of freedom	p value
WHO eTB (n = 108)	0.903	108	< 0.001
WHO TB (n = 109)	0.892	109	< 0.001

Table 5. Skewness test

Skewness	Statistic	Standard error
WHO eTB (n = 108)	-0.628	0.233
WHO TB (n = 109)	-0.404	0.231
